# Rare‐Earth–Sulfur Surface Modification Enables SiC Ceramics for Low‐Frequency Electromagnetic Wave Absorption in Extreme Environments

**DOI:** 10.1002/advs.76556

**Published:** 2026-07-08

**Authors:** Zhanming Wu, Xiaojun Zeng, Yu‐Nan Tan, Chi Yu, Nuohua Xie, Yanfeng Gao

**Affiliations:** ^1^ Jiangxi Key Laboratory of Advanced Ceramic Materials School of Materials Science and Engineering Jingdezhen Ceramic University Jingdezhen China; ^2^ State Key Laboratory of Advanced Refractories Shanghai University Shanghai China

**Keywords:** electromagnetic wave absorption, low‐frequency, polarization relaxation, rare‐earth, silicon carbide

## Abstract

The development of low‐frequency electromagnetic wave (EMW) absorbers with excellent stability in extreme environments remains a major challenge for radar stealth and anti‐electromagnetic interference applications. Herein, a rare‐earth–sulfur (RE─S) (RE = La, Ce, Pr, Sm, Gd, Er) surface modification strategy is first proposed to regulate the electromagnetic response of SiC‐based ceramics via surface chemical bond evolution from Si─O to RE─O species. This process transforms fast‐relaxation dipoles into slow‐relaxation dipoles, thereby prolonging the polarization relaxation time and inducing a low‐frequency shift in the absorption peak from the Ku band to the C band. The optimized SiC/Ce–S ceramics exhibits a minimum reflection loss (*R*_L_) of −60.08 dB at 5.76 GHz, while the strategy demonstrates broad universality across multiple RE elements. The enhanced EMW absorption performance is attributed to the synergistic regulation of surface chemistry, dipole polarization, and dielectric relaxation. Moreover, the RE─S modified ceramics show rapid thermal response, excellent corrosion resistance, and outstanding oxidation stability, retaining an *R*_L_ of −54.46 dB at 5.44 GHz after annealing at 500°C. This work provides a viable strategy for designing multifunctional SiC‐based EMW absorbers for operation in extreme environments.

## Graphical abstract

By altering surface chemical bonds to prolong relaxation time and shift absorption to lower frequencies, SiC/rare earth–sulfur (RE─S, RE = La, Ce, Pr, Sm, Gd, Er) materials achieve excellent low‐frequency electromagnetic wave absorption under extreme conditions, offering a new direction for designing high‐performance SiC‐based absorbers.

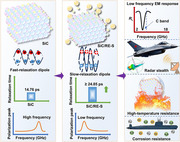

## 1 Introduction

With the rapid advancement of 5G communications technology, stealth submarines, and high‐speed vehicles, radar stealth and anti‐electromagnetic interference technologies have become focal points in both national defense security and civilian electronics [1, 2, 3]. In particular, the widespread deployment of 5G systems, together with the requirements for corrosion resistance in submarines and high‐temperature stability in hypersonic platforms, has significantly increased the demand for electromagnetic wave (EMW) absorption materials that operate efficiently at low frequencies (C band and below) in extreme environments [4, 5, 6].

Traditionally, magnetic metals (e.g., Fe, Co, Ni) and their alloys exhibit excellent low‐frequency EMW absorption performance owing to their high saturation magnetization and permeability, which generate strong natural resonance and eddy current loss [7, 8]. However, their practical application in extreme environments is severely limited by low Curie temperatures and poor corrosion resistance. The low Curie temperature results in significant attenuation or even complete loss of magnetic moments at elevated temperatures, while inadequate corrosion resistance leads to rapid material degradation in humid or saline environments. Both factors markedly deteriorate their low‐frequency EMW absorption performance [9, 10]. Consequently, conventional magnetic metals are unsuitable for high‐temperature and corrosive service environments.

Silicon carbide (SiC), with high thermal stability, corrosion resistance, and tunable dielectric properties, shows considerable potential for EMW absorption in extreme environments [11, 12]. However, pristine SiC suffers from intrinsically limited dielectric loss capability and short polarization relaxation times (*τ*), resulting in limited EMW absorption performance in the low‐frequency region [13, 14]. Therefore, a key scientific challenge about SiC ceramics is to achieve a controllable shift of the absorption peak from high to low frequencies through non‐magnetic mechanisms, while maintaining structural and functional stability in extreme environments.

To address this issue, various strategies have been explored to enhance the low‐frequency EMW absorption of SiC ceramics. For example, Wei et al. [15]. constructed hierarchical porous SiC decorated with in situ grown nanowires, achieving a reflection loss (*R*_L_) of −64.98 dB at 7.8 GHz. Li et al. [16]. prepared SiC/Si_3_N_4_ composite nanowires via component hybridization, achieving a *R*_L_ of −48 dB at 6.5 GHz. Although these methods have enhanced low‐frequency EMW absorption performance, most existing strategies primarily focus on tuning dielectric constants through structural or compositional design, while overlooking the intrinsic relationship between surface chemical states and low‐frequency dielectric response [17, 18, 19]. Consequently, the underlying low‐frequency response mechanism remains unclear.

In this work, we propose a rare‐earth–sulfur (RE─S) (RE = La, Ce, Pr, Sm, Gd, Er) surface modification strategy to address the limited low‐frequency EMW absorption performance of SiC by directly regulating its surface chemical environment. In this approach, RE ions function as “oxygen traps” during high‐temperature sulfidation, promoting the dissociation of Si─O bonds and the formation of RE─O polarization centers. This process enables the transformation of polarization units from fast‐relaxation dipoles to slow‐relaxation dipoles, thereby providing a new pathway for tuning polarization relaxation without relying on magnetic loss. The optimized SiC/Ce─S ceramics exhibit a *R*_L_ of −60.08 dB at low‐frequency of 5.76 GHz, while simultaneously demonstrating rapid thermal response, excellent corrosion resistance, and outstanding high‐temperature oxidation stability. Compared with conventional structural or compositional optimization strategies, this approach establishes a direct correlation between surface chemical bonding and dielectric relaxation behavior. Consequently, this strategy enables low‐frequency EMW absorption while maintaining structural stability in extreme environments, making it suitable for applications in aerospace systems, marine environments, and high‐power electronic devices.

## 2 Results and Discussion

**FIGURE 1:**
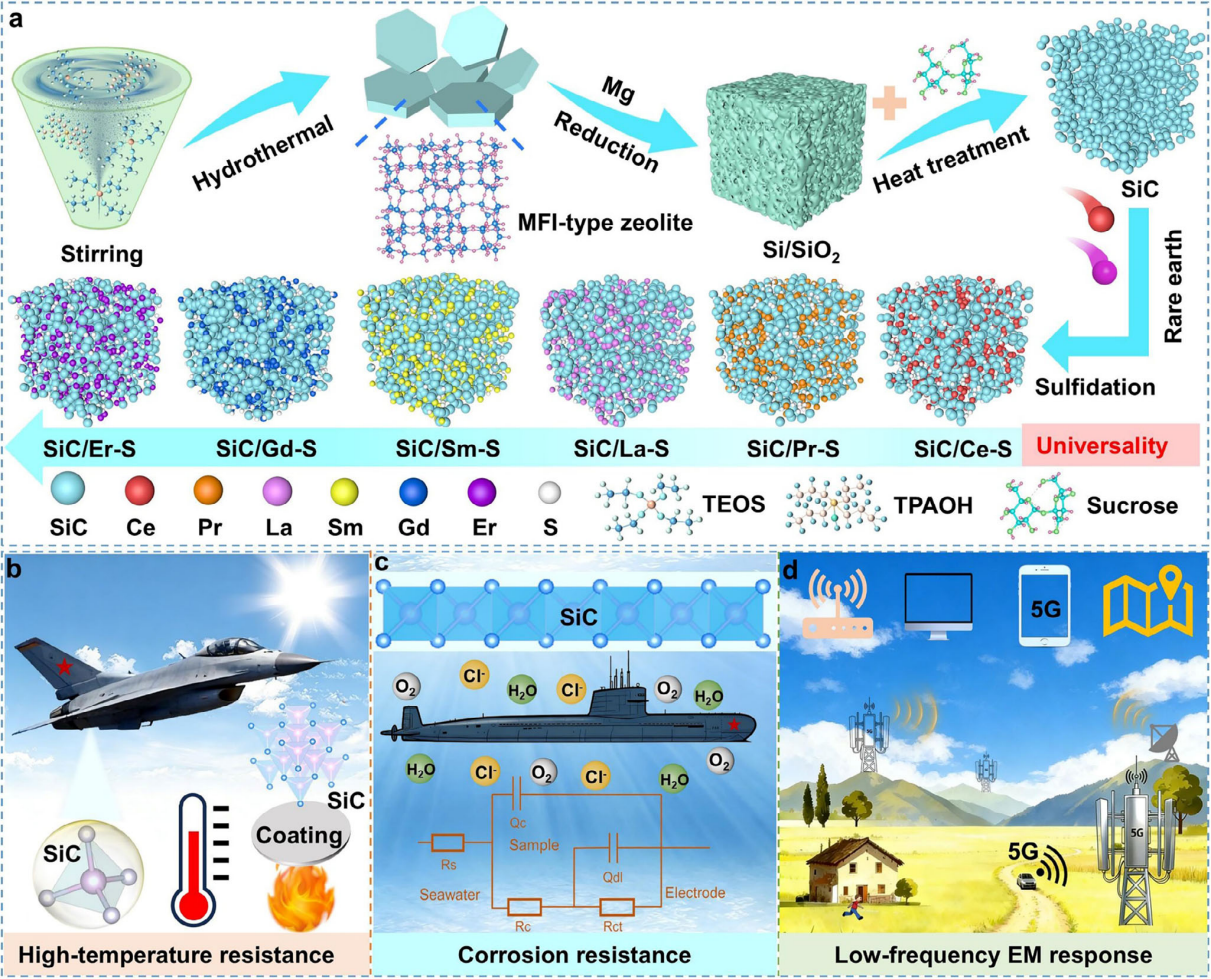
(a) Schematic illustration of the synthesis process for SiC/RE─S ceramics. (b–d) Depictions of the multifunctional applications for SiC/RE─S ceramics.

Figure 1 schematically illustrates the synthesis process of SiC/RE─S (RE = La, Ce, Pr, Sm, Gd, Er) ceramics. First, an MFI‐type zeolite precursor with an ordered mesoporous structure is synthesized via a hydrothermal method using tetrapropylammonium hydroxide (TPAOH) as the structure‐directing agent and tetraethyl orthosilicate (TEOS) as the silicon source. Subsequently, the as‐obtained zeolite is subjected to a magnesiothermic reduction, wherein Mg vapor reduces the highly reactive silicon species within the zeolite framework in situ, forming a Si/SiO_2_ composite. This resulting intermediate is then thoroughly mixed with sucrose as the carbon source, followed by drying and a high‐temperature carbothermal reduction to generate SiC with residual oxygen‐containing polar bonds (e.g., Si─O) on the surface. The resulting SiC is further dispersed in a RE nitrate solution and subjected to a high‐temperature sulfidation treatment. During this process, the reducing atmosphere generated by the decomposition of thiourea (e.g., H_2_S, CS_2_) synergistically interacts with the RE ions, promoting the etching and partial substitution of surface Si─O bonds. Simultaneously, RE species with strong oxophilicity preferentially form RE─O bonds anchored on the SiC surface. This surface modification effectively reduces the density of polar Si─O bonds while introducing high‐mass and large‐radius RE‐based dipolar centers. As a result, the dielectric properties and polarization relaxation behavior can be systematically tuned through regulation of the surface chemical environment. Consequently, the as‐fabricated SiC/RE─S ceramics exhibit high‐temperature resistance, corrosion resistance, and improved low‐frequency electromagnetic (EM) response (Figure 1b–d).

**FIGURE 2:**
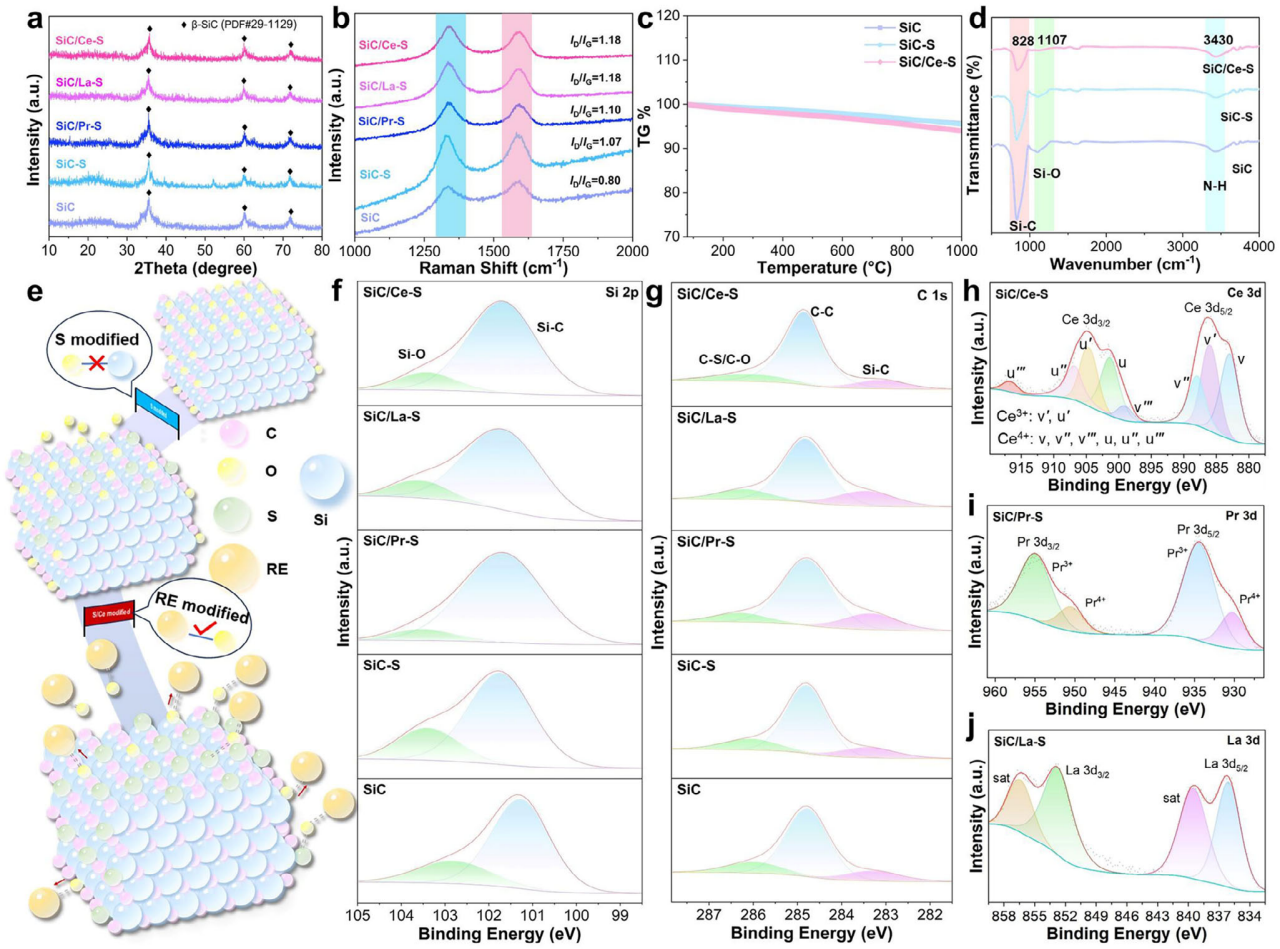
(a) XRD patterns and (b) Raman spectra of SiC, SiC─S, SiC/Ce─S, SiC/La─S, and SiC/Pr─S. (c) TG curves, (d) FT‐IR spectra, and (e) schematic illustration of the chemical bond evolution for SiC, SiC─S, and SiC/RE─S. High‐resolution XPS spectra of (f) Si 2p, (g) C 1s, (h) Ce 3d, (i) Pr 3d, and (j) La 3d for SiC, SiC─S, SiC/Ce─S, SiC/La─S, and SiC/Pr─S.

The phase composition and structural evolution of the as‐synthesized ceramics were characterized by x‐ray diffraction (XRD). As shown in Figure 2a and Figure S1, all samples (SiC, SiC─S, and SiC/RE─S) exhibit sharp diffraction peaks at 2*θ*≈35.7°, 60.0°, and 71.9°, corresponding to the (111), (220), and (311) crystal planes of *β*‐SiC (PDF#29‐1129), respectively. This confirms the successful formation of a highly crystalline SiC phase via carbothermal reduction [20]. Raman spectroscopy (Figure 2b) reveals the characteristic D band (∼1350 cm^−1^) and G band (∼1580 cm^−1^), associated with disordered and graphitic carbon, respectively. The defect level was evaluated by the intensity ratio (*I*_D_/*I*_G_) obtained from peak fitting [21]. Compared with pristine SiC, both SiC─S and SiC/RE─S exhibit higher *I*_D_/*I*_G_ values, indicating an increased defect density induced by sulfidation and RE incorporation. Thermogravimetric (TG) analysis was conducted to evaluate the thermal stability of the samples (Figure 2c). Upon heating from room temperature to 1000°C under argon atmosphere, all samples (SiC, SiC─S, and SiC/Ce─S) show gradual mass loss without abrupt degradation. The residual mass at 1000°C remains high, reaching 95.4% for SiC, 95.6% for SiC─S, and 94.0% for SiC/Ce─S, demonstrating that the SiC framework maintains excellent structural integrity after RE–S modification.

The evolution of surface chemical bond was further probed by Fourier transform infrared (FT‐IR) spectroscopy (Figure 2d,e). All samples exhibit a strong absorption band at 828 cm^−1^, which can be attributed to the Si─C stretching vibration of *β*‐SiC [12]. A key feature is the band at 1107 cm^−1^, assigned to Si─O bonds, which reflects the presence of oxygen‐containing surface groups [22]. For pristine SiC, this peak is notably intense, indicating abundant surface Si─O (e.g., Si─OH and Si─O─Si) species formed during carbothermal processing. After sulfidation (SiC─S), the intensity of the Si─O peak at 1107 cm^−1^ decreases, suggesting partial substitution of Si─O bonds by sulfur‐containing species (e.g., Si─S or C─S), driven by reactions with reductive gases (H_2_S, CS_2_) generated from thiourea decomposition. However, the persistence of the Si─O peak indicates incomplete substitution, consistent with thermodynamic limitations. Upon the introduction of RE element, the Si─O characteristic peak is significantly reduced, indicating a modification occurring on the SiC surface. According to the Hard and Soft Acid‐Base (HSAB) theory [23], RE^3+^ ions are typical hard acids with strong affinity for O^2−^. During high‐temperature process, the RE ions act as effective oxygen scavengers, preferentially capturing oxygen released from Si─O bond cleavage and forming stable RE─O species in situ. This process shifts the sulfur–oxygen substitution equilibrium and further promotes the dissociation of Si─O bonds. Consequently, the dominant polarization centers evolve from fast‐relaxation Si─O dipoles to slower‐relaxation RE─O species, effectively increasing the relaxation time [24].

X‐ray photoelectron spectroscopy (XPS) was employed to further analyze the elemental composition and chemical states. As shown in Figure S2, Si, C, O, S, N, and RE elements are detected, with relatively low contents of RE and S, thereby precluding the formation of crystalline RE oxysulfides or sulfides. In the high‐resolution XPS spectrum of Si 2p (Figure 2f), pristine SiC exhibits two peaks at 101.7 and 103.4 eV, corresponding to Si─C and Si─O bonds, respectively [25]. After RE modification, the Si–O peak shows a slight shift and decreased intensity, indicating changes in the local chemical environment. The C 1s spectra (Figure 2g) can be deconvoluted into three characteristic peaks at 283.3, 284.8, and 286.0 eV, corresponding to Si─C, C─C, and C─S/C─O bonds, respectively [26]. The Ce 3d and Pr 3d spectra reveal the coexistence of Ce^3^^+^/Ce^4^^+^ and Pr^3^^+^/Pr^4^^+^ (Figure 2h,i) [27], while the La 3d spectrum (Figure 2j) shows characteristic doublet peaks at 836.0 eV (La 3d_5/2_) and 852.9 eV (La 3d_3/2_), accompanied by satellite features, confirming the presence of La^3^^+^ species [28]. These results verify the interaction between RE elements and surface oxygen species. To investigate the evolution of surface oxygen chemical states, we performed high‐resolution O 1s XPS analysis (Figure S3). Pristine SiC exhibits a single peak at 532.5 eV, assigned to Si─O bonds. After sulfidation without RE addition, no significant change is observed. In contrast, following the introduction of RE, a new peak attributed to RE─O species emerged at 531.5 eV, while the intensity of the Si─O peak decreased significantly [29]. This indicates that RE ions actively capture oxygen atoms from dissociated Si─O bonds to form RE─O polarization centers, thereby acting as efficient ‘oxygen traps’.

**FIGURE 3:**
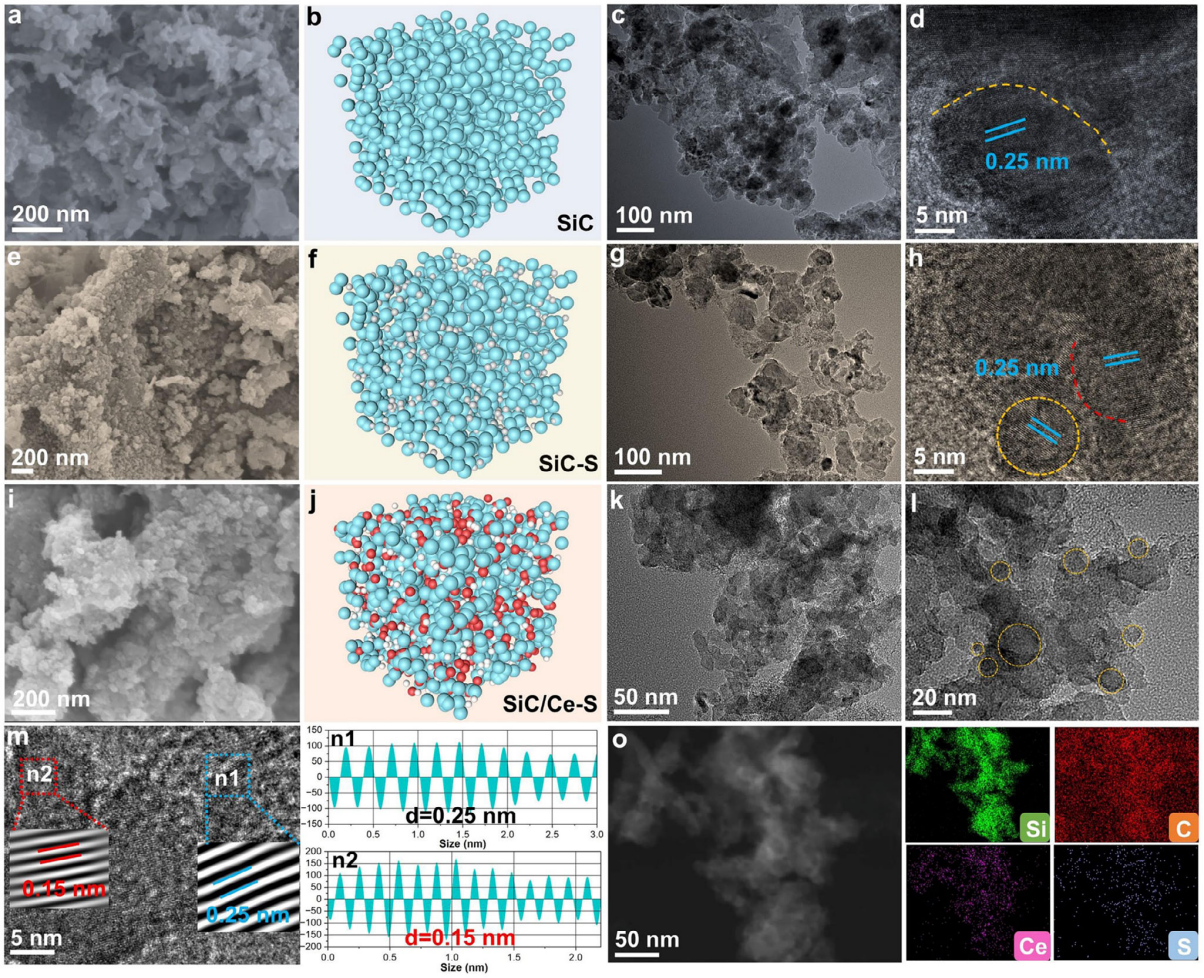
(a,e,i) SEM, (b,f,j) model, (c,g,k) TEM, and (d,h,l,m,n) HRTEM images of (a–d) SiC, (e–h) SiC─S, and (i–n) SiC/Ce─S. (o) EDS mapping images of SiC/Ce─S.

The microstructure and crystal structure of SiC, SiC─S, and SiC/Ce─S are presented in Figure 3. Scanning electron microscopy (SEM) image reveals a 3D architecture composed of densely interconnected nanoparticles (Figure 3a). This loosely packed structure facilitates subsequent surface modification. The corresponding structural model is illustrated in Figure 3b. Transmission electron microscopy (TEM) image further confirms that the particle sizes distributed within the nanometer range (Figure 3c). High‐resolution TEM (HRTEM) image exhibits well‐resolved lattice fringes with an interplanar spacing of 0.25 nm (Figure 3d), corresponding to the (111) plane of the *β*‐SiC, indicating the successful formation of a highly crystalline SiC substrate [30]. Figure 3e shows that the sulfidation treatment does not alter the original nanoparticle‐assembled morphology of SiC (Figure 3f). Figure 3g,h reveal that the primary crystalline phase remains SiC after sulfidation, with clearly observable lattice fringes corresponding to the (111) plane with a d‐spacing of 0.25 nm. These results indicate that sulfur species are primarily introduced through surface bonding or functional group substitution, rather than forming new crystalline phases.

Figure 3i demonstrates that the nanoparticle‐assembled framework is well preserved after RE impregnation and subsequent sulfidation (Figure 3j). Uniform elemental distribution can also be observed from the energy‐dispersive x‐ray spectroscopy (EDS) mapping image (Figure S4). Figure 3k,l further confirm the retained particle‐assembled morphology. Figure 3m,n show the lattice spacings of 0.25 and 0.15 nm, corresponding to the (111) and (220) planes of *β*‐SiC, respectively [30]. Importantly, Figure 3o and Figure S4 reveal the homogeneous distribution of Si, C, Ce, S, and O elements throughout the sample, with spatial profiles consistent that closely match the observed morphology. This provides the successful modification of the SiC surface with Ce─S elements. Additionally, SEM, TEM, and corresponding EDS analyses of SiC/La─S and SiC/Pr─S exhibit similar morphologies, crystal structures, and uniform elemental distributions (Figures S5–S9). Therefore, the designed stepwise modification strategy successfully achieves uniform loading of RE and S elements onto the SiC nanoparticle surface while preserving the structural framework.

**FIGURE 4:**
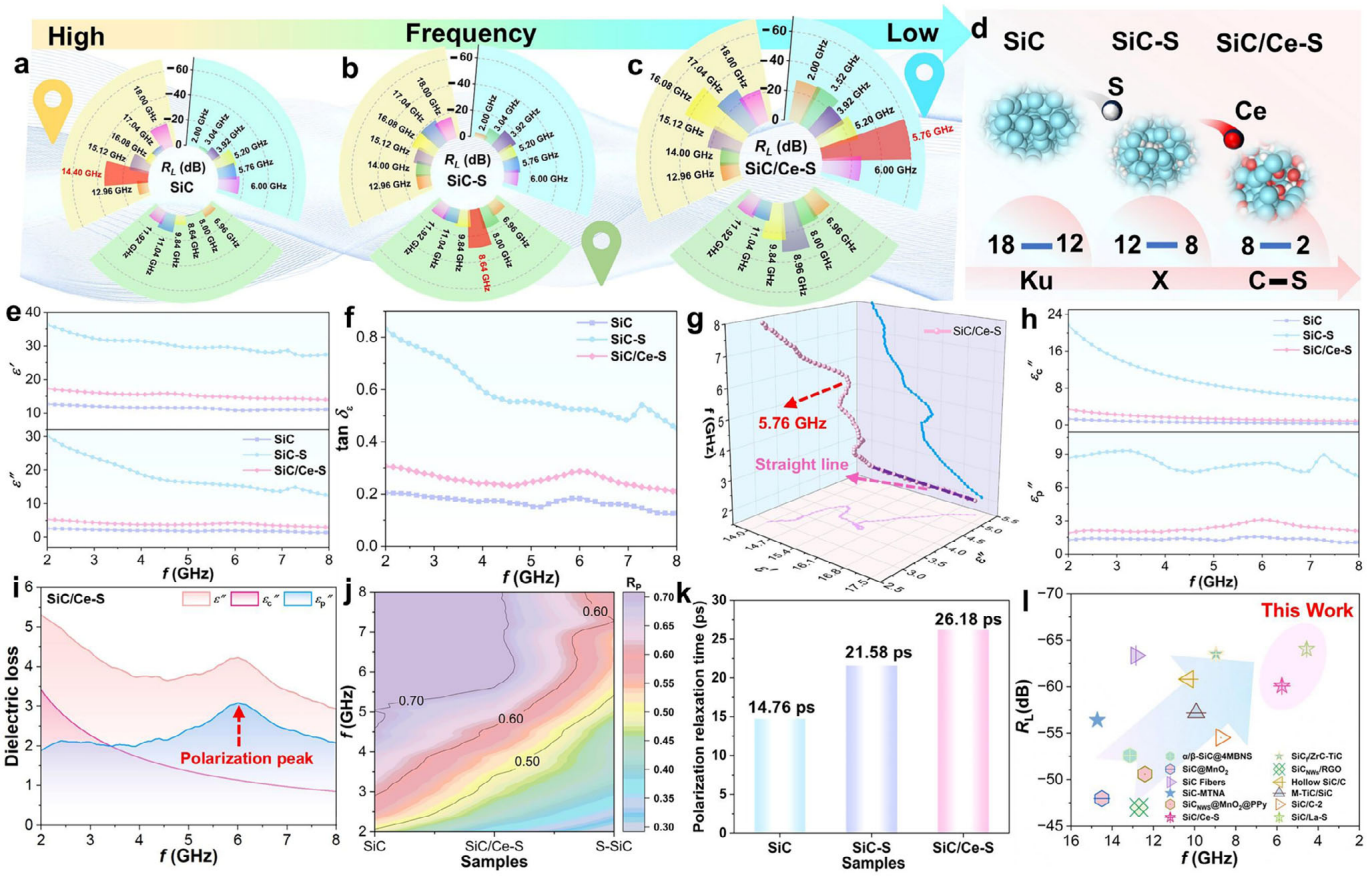
(a–c) *R*_L_ contour maps from high frequency to low frequency. (d) Schematic illustration of frequency shift. (e) *ε′*, *ε″*, and (f) tan *δ*_ε_ values of the samples. (g) Cole–Cole plots of SiC/Ce─S. (h) *ε*_c_ *″* and *ε*_p_ *″* of the samples. (i) Decomposition of *ε*_p_ *″* for SiC/Ce─S. (j) Polarization loss proportion of the samples. (k) *τ* value of the samples. (l) Comparison of EMW absorption performance of SiC/Ce─S with reported advanced SiC‐based ceramics.

The evolution of EMW absorption performance of SiC, SiC─S, and SiC/Ce─S ceramics is summarized in in Figure 4a–c and Figure S10. The pristine SiC ceramic exhibits a reflection loss (*R*_L_) of −34.62 dB at 14.4 GHz (Ku‐band) is achieved at a thickness of 4.99 mm, indicating that its EMW absorption is predominantly concentrated in the high‐frequency region (Figure 4a). After sulfur modification, the strongest absorption peak of the SiC─S shifts to 8.64 GHz (X‐band) with a *R*_L_ of −28.77 dB at a matching thickness of 3.20 mm (Figure 4b), suggesting that the introduction of sulfur effectively modulates the dielectric response of SiC. Notably, upon further introduction of cerium, SiC/Ce─S achieves a *R*_L_ of −60.08 dB at 5.76 GHz (C‐band) is obtained at a thickness of 3.51 mm. (Figure 4c), realizing a low‐frequency shift from the Ku‐band to the C‐band (Figure 4d). Figure 4e presents the frequency‐dependent variations of the real part (*ε′*) and imaginary part (*ε″*) of the complex permittivity (*ε*_r_). Pristine SiC exhibits the lowest permittivity, with *ε′* showing a gradual decreasing from a value of 12.5 at 2 GHz. However, the further introduction of cerium and sulfur increases *ε′* to 17.3 at 2 GHz for SiC/Ce─S. The variation trend of the dielectric loss tangent (tan *δ_ε_* = *ε″*/*ε′*) is highly consistent with that of the complex permittivity (Figure 4f). According to the Debye relaxation theory, the relationship between *ε*′ and *ε*″ can be expressed as (*ε*′−*ε*_∞_)^2^+(*ε*″)^2^ = (*ε*_s_−*ε*_∞_)^2^ [31]. The Cole–Cole plot of the SiC/Ce─S exhibits a prominent semicircle centered around 5.76 GHz (Figure 4g), which corresponds to a Debye relaxation process and further confirms the presence of polarization loss [32]. Additionally, a linear tail appears in the low‐frequency region (S‐band), signifying the contribution of conduction loss [33]. Multiple semicircles and linear tails are also observed for SiC and SiC─S (Figure S11).

To further elucidate the EMW loss mechanism, the total dielectric loss is deconvoluted into conduction loss and polarization loss components (Figure 4h), with detailed procedures provided in the Equations S1 and S2. As shown in Figure 4i, for SiC/Ce─S, conduction loss slightly dominates in the low‐frequency range (2–3.5 GHz), whereas polarization loss becomes the primary contributor above 3.5 GHz, with a pronounced relaxation peak near 6 GHz. For all samples, the contribution of polarization loss increases with frequency, while conduction loss gradually decreases (Figure 4j and Figure S12), which indicates that the polarization loss plays a dominant role in EMW attenuation. The position of the relaxation peak is related to the relaxation time (*τ*), defined as *τ* = 1/(2*πf*_m_), where *f*_m_ is the characteristic frequency of the dominant relaxation peak for each sample [34, 35]. SiC exhibits a relatively short *τ* of 14.76 ps, which increases to 21.58 ps for SiC─S and further to 26.18 ps for SiC/Ce─S (Figure 4k and Figure S13). The prolonged *τ* implies a slower polarization relaxation response to the alternating electromagnetic field, which is favorable for energy dissipation in the low‐frequency range, in agreement with the experimentally observed shift of the *R*_L_ peak toward lower frequencies [36].

The attenuation constant (*α* = [((2)^1/2^π*f*)/*c*]{(*µ“ε”*−*µ'ε'*)+[(*µ“ε”*−*µ'ε'*)^2^+(*ε'µ“*+ *ε”µ'*)^2^]^1/2^}^1/2^) and impedance matching ratio (*Z* = Z_in_/Z_0_) are critical for achieving excellent EMW absorption performance [37]. The *α* value reflects the intrinsic energy dissipation capability, while *Z* value determines the efficiency of EMW entry into the absorber [38]. A higher *α* value corresponds to stronger attenuation. However, effective absorption also requires appropriate *Z* value. As shown in Figure S14, although SiC─S shows the highest *α* value, its *R*_L_ remains relatively high due to impedance mismatch caused by its excessively high permittivity [39]. In contrast, SiC/Ce─S ceramics achieve a balanced combination of moderate attenuation capability and favorable impedance matching, enabling superior EMW absorption performance. Compared with recently reported typical SiC‐based ceramics (Figure 4l), the SiC/Ce─S ceramics demonstrate significant advantages in low‐frequency EMW absorption performance.

**FIGURE 5:**
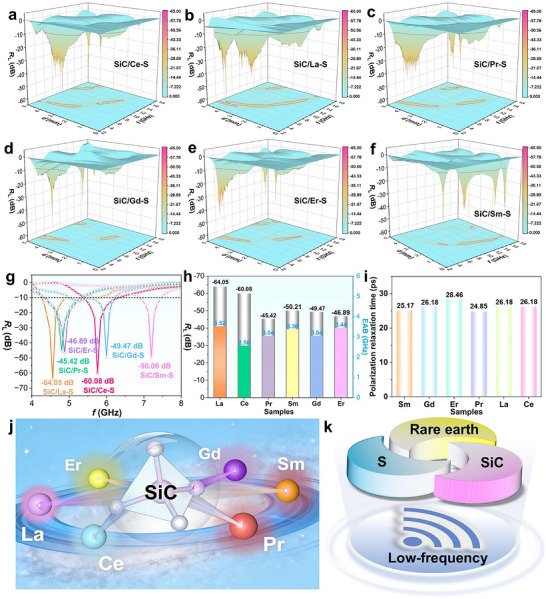
(a–f) 3D *R*_L_ plots of SiC/RE─S. (g) 2D *R*_L_ plots in the 4–8 GHz range of SiC/RE─S. (h) Statistical bar chart of *R*_L_ and EAB. (i) *τ* values of SiC/RE─S. (j) The universal EMW absorption performance across different RE elements. (k) The synergistic interaction among SiC, sulfur, and RE elements that enables low‑frequency EMW absorption.

To verify the universality of the synergistic effect, the SiC modified with different RE (Ce, La, Pr, Sm, Gd, and Er) and sulfur elements was systematically investigated in the low‐frequency EMW absorption. SiC/Ce─S achieves a *R*_L_ of −60.08 dB at 5.76 GHz (Figure 5a), while SiC/La─S exhibits the best performance with a *R*_L_ of −64.05 dB at 4.56 GHz (Figure 5b). SiC/Pr─S shows a *R*_L_ of −45.42 dB at 4.8 GHz (Figure 5c), SiC/Sm─S reaches −50.06 dB at 7.2 GHz (Figure 5d), SiC/Gd─S achieves −49.47 dB at 6.0 GHz (Figure 5e), and SiC/Er─S delivers −46.89 dB at 4.88 GHz (Figure 5f). Notably, all SiC/RE─S samples exhibit *R*_L_ values below −45 dB, with absorption peaks concentrated in the low‐frequency (4–8 GHz) region. This behavior contrasts sharply with pristine SiC (14.4 GHz) and SiC─S (8.6 GHz), confirming that the synergistic effects of RE and sulfur elements is the key factor driving the low‐frequency shift of the absorption peak. Figure 5g presents an overlay comparison of the 2D *R*_L_ curves for the samples, demonstrating the commonality of strong EMW absorption performance in the low‐frequency region for all RE–S modification samples. Figure 5h summarize the comparison of effective absorption bandwidths (EAB) for each sample. Among them, SiC/La─S exhibits the widest EAB of 3.52 GHz, while the bandwidths of the remaining samples all exceed 2.56 GHz.

As shown in Figure S15a–c, the real and imaginary parts of the permittivity, as well as the dielectric loss of SiC/RE─S, are all higher than those of SiC. Furthermore, a comparative analysis of the subdivided conduction loss and polarization loss reveals that SiC/RE─S remains dominated by polarization loss, with their polarization peaks located at low frequencies (Figure S15d,e) [40]. *τ* calculated based on the peak positions range from 24.8 to 28.5 ps, all significantly higher than that of SiC (Figure 5i). This enhancement is consistently observed across the RE series from light (La) to heavy (Er) elements, demonstrating the universality of the RE─S modification strategy (Figure 5j,k). The Cole–Cole plots of SiC/RE─S exhibit multiple semicircles, indicating pronounced polarization loss (Figure S16) [41]. As shown in Figure S17, the SiC/RE─S samples exhibit favorable attenuation capability, while their *Z* values at low frequencies are all close to 1, indicating efficient electromagnetic wave entry into the material. Overall, these results demonstrate that the RE–S surface modification strategy is effective in tuning dielectric behavior, prolonging polarization relaxation, and enhancing low‐frequency EMW absorption performance in SiC‐based systems.

**FIGURE 6:**
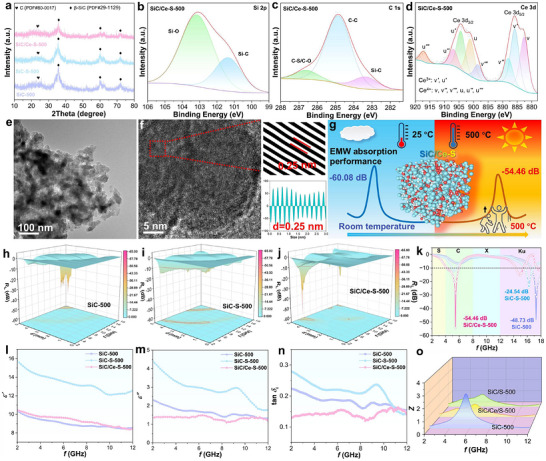
(a) XRD patterns of SiC‐500, SiC─S‐500, and SiC/Ce─S‐500. (b–d) High‐resolution XPS spectra of Si 2p, C 1s, and Ce 3d for SiC/Ce─S‐500. (e,f) TEM images of SiC/Ce─S‐500. (g) Schematic of EMW absorption performance before and after thermal treatment at 500°C. (h–j) 3D *R*_L_ plots, (k) 2D *R*_L_ plots, (l) *ε′*, (m) *ε″*, (n) tan *δ*_ε_, and (o) *Z* values of SiC‐500, SiC─S‐500, and SiC/Ce─S‐500.

To evaluate EMW absorption performance in extreme environments, the structural evolution and electromagnetic response of SiC/Ce─S after oxidation at 500°C in air (denoted as SiC/Ce─S‐500) were systematically investigated. Pristine SiC and SiC─S were examined for comparison to elucidate the role of RE–S modification in enhancing high‐temperature stability. XRD pattern of SiC/Ce─S‐500 is consistent with that before oxidation, with no discernible signals from SiO_2_ or other crystalline oxidation products, indicating that the main crystalline phase of the ceramics remains intact after high‐temperature oxidation (Figure 6a). However, the XPS survey spectrum and the corresponding elemental content (Figure S18) reveal an increase in oxygen content after oxidation. The high‐resolution Si 2p XPS spectrum in Figure 6b shows a significantly increased relative intensity of the Si─O bond (103.5 eV), even surpassing that of the Si─C bond (101.7 eV), confirming the formation of an amorphous SiO_2_ layer on the surface. In the C 1s spectrum (Figure 6c), peaks corresponding to Si─C (283.4 eV), C─C (284.8 eV), and C─O/C─S (286.6 eV) are still observed, indicating the preservation of surface functional groups. The Ce 3d spectrum (Figure 6d) exhibits complex multiplet splitting characteristics, indicating that the coexistence of Ce^3+^ and Ce^4+^, consistent with the pre‐oxidation state [42]. The TEM image in Figure 6e shows that the nanoparticle‐assembled morphology is retained after oxidation. In the HRTEM image (Figure 6f), a lattice spacing of 0.15 nm corresponding to the (220) plane of SiC is clearly resolved, demonstrating that the internal crystal structure remains well preserved. A schematic comparison of EMW absorption performance before and after oxidation at 500°C is provided in Figure 6g. Pristine SiC‐500 exhibits a *R*_L_ of −48.74 dB (Figure 6h), while SiC─S‐500 shows a significant performance degradation (Figure 6i), with the *R*_L_ increasing to −24.54 dB. In contrast, SiC/Ce─S‐500 retains excellent EMW absorption performance (Figure 6j), achieving an *R*_L_ of −54.46 dB at 5.44 GHz. The corresponding 2D *R*_L_ comparison further confirms that the absorption peak of SiC/Ce–S‐500 remains in the C band with minimal attenuation in intensity (Figure 6k). Figure 6l–n displays the *ε′*, *ε″*, and tan *δ*_ε_ values of the three samples after oxidation, respectively. Compared with their pre‐oxidation states, all samples exhibit a decrease in permittivity, attributing to the surface formation of amorphous SiO_2_ [43]. Figure 6o shows that SiC/Ce─S‐500 exhibits *Z* value closest to 1 near 5.4 GHz. This optimization arises from the surface SiO_2_ thin layer effectively balancing the impedance at the air–material interface. As shown in Figure S19, SiC/Ce─S‐500 maintains a relatively high attenuation constant in the low‐frequency region, indicating preserved dissipation capability. Furthermore, the Cole–Cole plots confirm that polarization relaxation processes are still active after oxidation (Figure S20). After oxidation at 500°C, the surface‐formed SiO_2_ and the stable internal structure promotes the strong EMW absorption performance. To further evaluate the high‐temperature oxidation resistance of the SiC/Ce─S ceramics, we raised the heat treatment temperature to 800°C (in air). Following oxidation at 800°C, the sample maintained a *R*_L_ of −25.56 dB at 6.8 GHz (C‐band) (Figure S21), indicating that the material retains effective low‐frequency absorption capabilities at this temperature.

**FIGURE 7:**
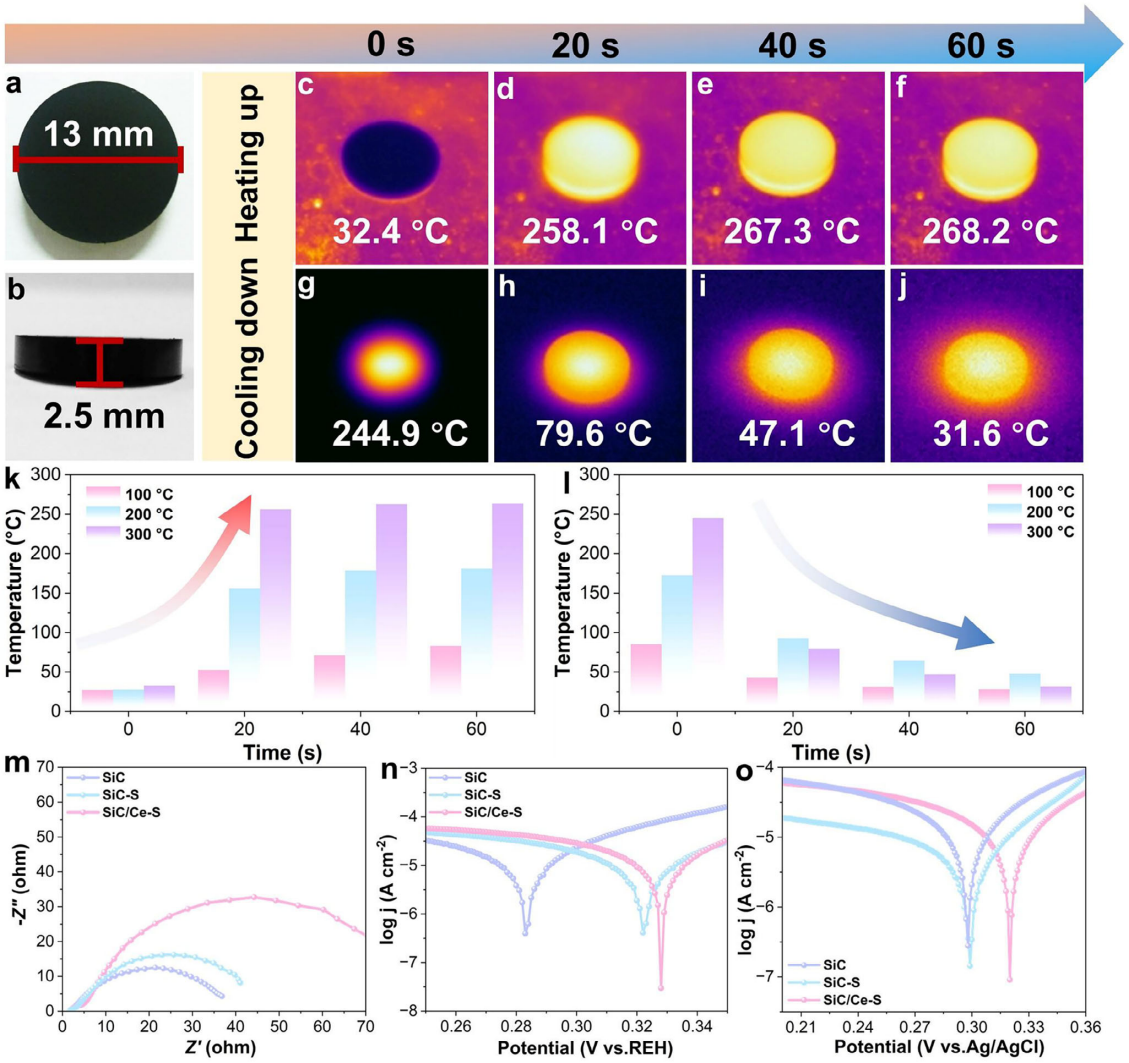
(a,b) Diameter and thickness of the pressed SiC/Ce─S pellets. (c–f) Infrared thermal images of the heating process for SiC/Ce─S. (g–j) Infrared thermal images of cooling process for SiC/Ce─S. (k) Statistical plot of the heating process for SiC/Ce–S. (l) Statistical plot of the cooling process for SiC/Ce─S. (m) EIS (1 m KOH solution), (n) Tafel curves, and (o) Tafel curves (3 m NaCl solution) of SiC, SiC─S, and SiC/Ce─S.

To further investigate the thermal management capability of SiC/Ce─S, the powder was pressed into pellets with a diameter of 13 mm and a thickness of 2.5 mm under 10 MPa using a mold (Figure 7a,b) [44]. Figure 7c–f shows the thermal response that occurred after the sample was rapidly placed on a 300°C hot stage. The surface temperature increases rapidly to 258.1°C within 20 s, indicating a fast‐heating rate. Upon removal from the hot stage, the temperature decreases sharply from 244.9°C to 79.6°C within 20 s, demonstrating equally rapid cooling behavior (Figure 7g–j). To assess the robustness of thermal response, the tests were conducted at 100°C and 200°C hot stage (Figure S22). Figure 7k,l further presents quantitative bar charts of the heating and cooling rates at 100°C, 200°C, and 300°C hot stage, respectively. The samples exhibit rapid heating and cooling, indicating excellent temperature sensitivity and thermal cycling stability [45]. For comparison, pristine SiC samples were tested under identical conditions. On a heating stage set to 300°C, the temperature of the pristine SiC rise from room temperature to 220.1°C within 20 s; after removal from the stage, it drops from 233.7°C to 143.2°C within 20 s (Figure S23). These results demonstrate that the SiC/Ce–S samples not only retain the excellent thermal management capabilities of the pristine SiC matrix but also exhibit enhanced thermal response characteristics. The excellent thermal response characteristics stem from the superior thermal management capabilities of SiC itself and the interconnected nanoparticle network; these factors synergistically facilitate rapid heat diffusion, a process unaffected by the Ce─S surface modification. The corrosion resistance of SiC, SiC─S, and SiC/Ce─S was further evaluated in different corrosive media using an electrochemical workstation [46]. Figure 7m shows the electrochemical impedance results in 1 mol·L^−1^ KOH solution. Clearly, SiC/Ce─S sample exhibits the largest arc radius, indicating the highest charge transfer resistance, which suggests a more superior corrosion resistance [47]. Tafel polarization curves measured in the same alkaline environment reveal that SiC/Ce─S possesses the most positive corrosion potential and the lowest corrosion current density (Figure 7n). In a simulated seawater environment of 3.5 wt.% NaCl (Figure 7o), SiC/Ce─S exhibits the most positive corrosion potential and lowest current density, demonstrating excellent corrosion resistance under marine environments [48]. The enhanced corrosion resistance can be attributed to the synergistic effect of RE─S modification, which reduces the density of Si─O polar bonds, thereby decreasing the surface affinity for polar corrosive media. Overall, the SiC/Ce─S ceramics exhibit outstanding thermal management capability and corrosion resistance, while maintaining excellent EMW absorption performance even after high‐temperature oxidation. These results demonstrate the robust stability of the SiC/Ce─S ceramics in extreme environments.

**FIGURE 8:**
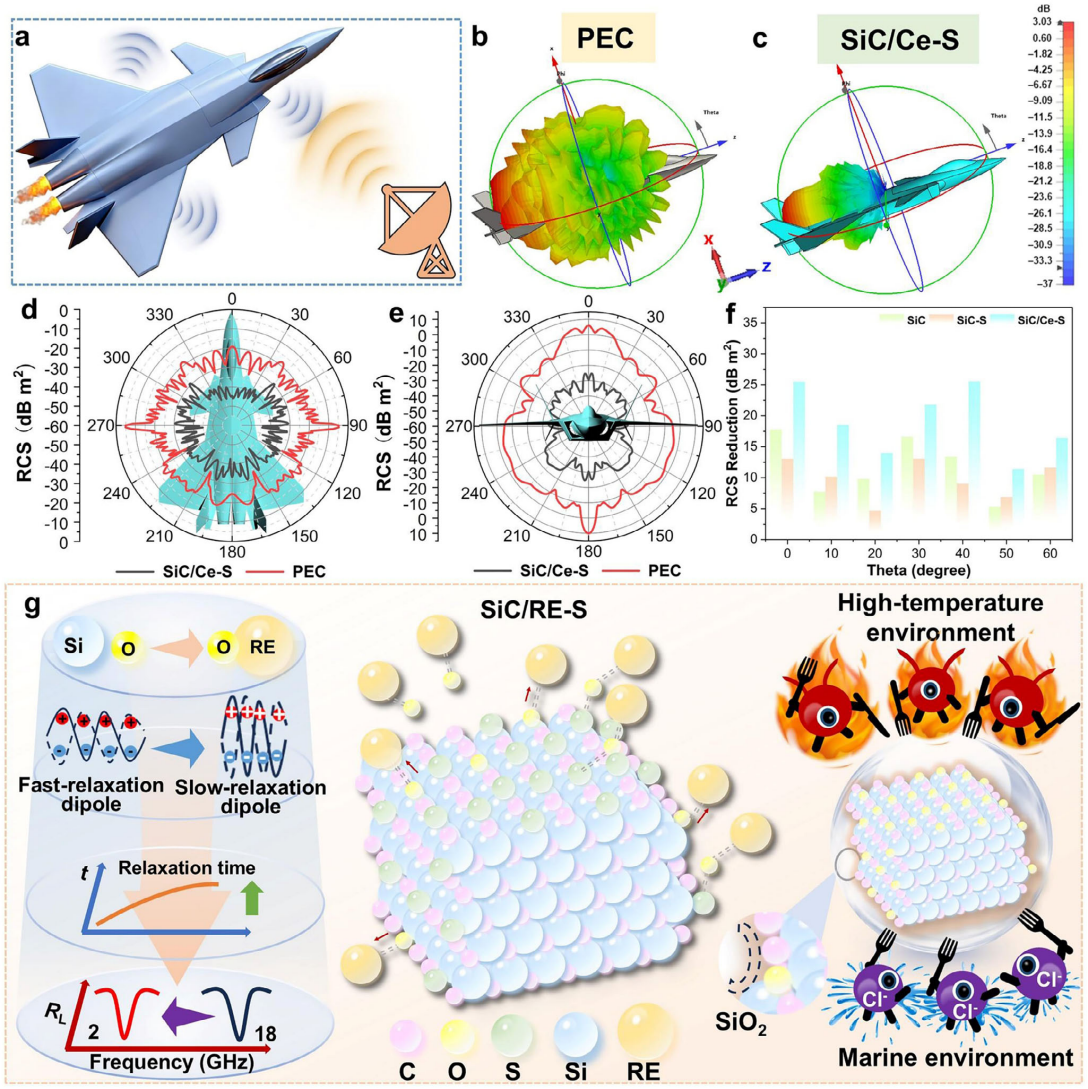
(a) Radar detection of the fighter models. (b,c) 3D RCS of fighter models of PEC and SiC/Ce─S. (d,e) 2D RCS curve of fighter models at top view and front view. (f) RCS values at different angles. (g) Schematic illustration of the EMW absorption mechanism for SiC/RE─S.

Radar stealth potential of SiC/Ce─S ceramics in practical scenarios was quantitatively evaluated by comparing the radar cross‐section (RCS) of the SiC/Ce─S‐coated model (coating thickness = 3.51 mm) with that of a perfect electric conductor (PEC) at 5.76 GHz. A simplified fighter aircraft model was constructed as stealth object using CST Microwave Studio 2024 (Figure 8a) [49]. Figure 8b shows the 3D RCS distribution of the PEC model, where strong specular reflections are observed at the nose, tail, wing leading edges, and normal fuselage regions. These areas correspond to high RCS values, indicating strong radar detectability. In contrast, after coating with SiC/Ce─S (Figure 8c), the scattering intensity across the entire surface is significantly reduced, and high‐reflection regions are markedly suppressed. At dominant scattering sites such as the nose and wings, the reflected signal is effectively attenuated, demonstrating excellent radar stealth performance [50]. Figure 8d,e presents the 2D RCS curves in the horizontal plane for the fuselage and nose sections, respectively. For both orientations, the SiC/Ce─S‐coated model exhibits consistently lower RCS values across all detection angles compared to the PEC reference. Notably, substantial reductions are observed at critical detection angles (e.g., near 0°), indicating effective suppression of radar signals from multiple directions [51]. Figure 8f further compares the RCS reduction (relative to PEC) of SiC, SiC─S, and SiC/Ce─S coatings over a detection angle range of 0°–60°. Pristine SiC and SiC─S show limited attenuation capability, whereas SiC/Ce─S demonstrates significantly enhanced performance across all angles, achieving a maximum RCS reduction of 25.56 dB·m^2^ at 40° [52]. These results confirm that the SiC/RE─S exhibits effective wide‐angle radar stealth in practical structural configurations.

Based on the above findings, a mechanism involving RE–S surface modification and polarization relaxation regulation is proposed to explain the low‐frequency and high‐efficiency EMW absorption in SiC‐based ceramics (Figure 8g). Pristine SiC contains abundant surface Si─O bonds, which act as fast‐relaxation dipoles that undergo dielectric relaxation primarily at high frequencies (Ku band), resulting in limited absorption intensity. After sulfur modification, the density of Si─O bonds are partially reduced. However, due to the relatively low bond strength of Si─S and thermodynamic constraints, this substitution remains incomplete. The key breakthrough lies in the introduction of RE elements. As typical hard acids, RE^3+^ ions exhibit a strong affinity for the hard base O^2−^. During the high‐temperature sulfidation process, the RE elements act as efficient “oxygen traps” capturing oxygen atoms dissociated from Si─O bonds to form thermodynamically stable RE─O bonds. This process disrupts the original sulfur–oxygen equilibrium, promoting continuous dissociation of Si─O bonds and changing the surface chemical environment. Consequently, the dominant polarization units evolve from fast‐relaxation Si─O dipoles to slow‐relaxation RE─O dipoles. Additionally, the RE─S modification introduces a substantial number of defects, including vacancies, dangling bonds, and RE─O clusters, which also serve as effective polarization centers, enhancing polarization loss under an alternating electric field. Consequently, the *τ* of SiC/RE─S is effectively prolonged, shifting the absorption peak from high to low frequencies. As a result, the RE─S surface modification strategy enables synergistic optimization of electromagnetic parameters through surface chemical bond evolution and polarization relaxation modulation.

## 3 Conclusions

In summary, a rare‐earth–sulfur (RE─S) surface modification strategy is proposed to regulate the electromagnetic response of SiC‐based ceramics through surface chemical bond evolution. The synergistic interaction between RE elements and sulfur promotes the directional dissociation of surface Si─O bonds and the in situ formation of RE─O species. This transformation converts fast‐relaxation Si─O dipoles into slow‐relaxation RE─O polarization centers, thereby effectively prolonging the relaxation time and enabling a shift of the polarization relaxation from high to low frequencies. As a representative system, SiC/Ce─S exhibits a *R*_L_ of −60.08 dB in the low‐frequency region. Notably, this strategy demonstrates excellent universality, as SiC/RE–S ceramics modified with different RE elements (La, Ce, Pr, Sm, Gd, and Er) achieve stable absorption peaks in the C band with *R*_L_ values exceeding −45 dB. In addition, the ceramics exhibit rapid thermal response, excellent corrosion resistance, and outstanding high‐temperature oxidation stability. Even after oxidation at 500°C, SiC/Ce─S‐500 retains strong EMW absorption performance (*R*_L_ = −54.46 dB at 5.44 GHz). Radar cross‐section (RCS) simulations further demonstrate a maximum attenuation of 25.56 dB·m^2^, highlighting the potential for practical radar stealth applications. Overall, this work establishes a relationship between chemical bond evolution and electromagnetic response, offering a promising pathway for the design of low‐frequency EMW absorbers capable of operating in extreme environments.

## 4 Experimental

### 4.1 Chemicals

Tetraethyl orthosilicate (TEOS), sucrose (C_12_H_22_O_11_, 99%), thiourea (CH_4_N_2_S, 99%), and hydrochloric acid (HCl, 36 wt.%) were purchased from Sinopharm Chemical Reagent Co., Ltd. Tetrapropylammonium hydroxide (TPAOH, 25 wt.%) was obtained from Anhui Senrise Technologies Co., Ltd. Magnesium powder (Mg, 99.5%) was supplied by Tianjin Damao Chemical Reagent Factory. Rare‐earth nitrates, including Ce(NO_3_)_3_·6H_2_O, Pr(NO_3_)_3_·6H_2_O, La(NO_3_)_3_·6H_2_O, Gd(NO_3_)_3_·6H_2_O, Sm(NO_3_)_3_·6H_2_O, and Er(NO_3_)_3_·5H_2_O, were obtained from Shanghai Macklin Biochemical Co., Ltd. All chemicals were used as received without further purification.

### 4.2 Synthesis of MFI‐Type Silicon Zeolites

MFI‐type zeolites were synthesized via hydrothermal method. Specifically, 6.93 g of TEOS, 10.56 g of TPAOH (25 wt.%), and 15.84 g of deionized water were mixed and stirred at room temperature for 8 h. The resulting homogeneous solution was transferred into a Teflon‐lined autoclave and heated at 180°C for 48 h. The resulting solid was collected, washed with deionized water and ethanol to neutral pH, dried at 100°C for 12 h, and calcined at 550°C for 4 h in air.

### 4.3 Preparation of Si/SiO_2_ Composites

Mesoporous Si/SiO_2_ composites were prepared via magnesiothermic reduction. MFI‐type zeolite and Mg powder (mass ratio of 1:1) were ground for 45 min and then heated to 700°C (heating rate = 5°C·min^−1^) under an argon atmosphere for 5 h. The product was acid‐leached in 1 mol L^−1^ HCl for 24 h to remove MgO and Mg_2_Si, followed by centrifugation, repeated washing to neutral pH, and finally dried under vacuum at 80°C for 12 h to obtain Si/SiO_2_ powders.

### 4.4 Preparation of SiC Ceramics

Sucrose of 0.237 g was dissolved in 10 mL of deionized water, followed by the addition of Si/SiO_2_ powders (0.1 g). The suspension was ultrasonicated for 10 min and stirred for 3 h, then dried at 60°C for 12 h. The obtained precursor was heat‐treated under an argon atmosphere. The temperature was ramped to 800°C (heating rate = 2°C·min^−1^) and maintained for 2 h, followed by further heating to 1400°C (heating rate = 5°C·min^−1^) and maintained for 2 h to obtain SiC ceramics.

### 4.5 Preparation of SiC/RE–S Ceramics

SiC nanoparticles (100 mg) were dispersed in 10 mL of rare‐earth (RE) nitrate solution (0.02 mol·L^−1^) and stirred at 60°C for 6 h. After centrifugation, the precipitate was dried at 70°C to obtain RE‐loaded precursors. The precursors were mixed with thiourea (mass ratio of thiourea to precursor = 2:1) as the sulfur source and subjected to thermal treatment under an argon atmosphere. The temperature was increased to 600°C at a rate of 5°C·min^−1^ and maintained for 2 h to complete the sulfidation process. The resulting products were denoted as SiC/RE–S (RE = La, Ce, Pr, Sm, Gd, Er). A control sample (SiC─S) was prepared under identical conditions without RE salts.

To evaluate the thermal stability, SiC, SiC─S, and SiC/Ce─S samples were further annealed at 500°C for 1 h in an air atmosphere. The resulted samples were denoted as SiC‐500, SiC─S‐500, and SiC/Ce─S‐500, respectively.

### 4.6 Materials Characterization

The morphology of the samples was examined using field‐emission scanning electron microscopy (FESEM, SU‐8010, Hitachi, Japan) and transmission electron microscopy (TEM, JEM‐F200, Japan) equipped with energy‐dispersive x‐ray spectroscopy (EDS). Phase identification was carried out using x‐ray diffraction (XRD, D8 Advance, Bruker, Germany) with Cu Kα radiation. The graphitization degree of carbon was evaluated by Raman spectroscopy (LabRAM HR800, HORIBA, Japan). Surface chemical states and elemental composition were analyzed by x‐ray photoelectron spectroscopy (XPS, ESCALAB 250Xi, Thermo Fisher Scientific, USA). The Brunauer‐Emmett‐Teller (BET) specific surface area and pore size distribution were determined by nitrogen adsorption–desorption measurements using an analyzer (ASAP 2020 M, Micromeritics, USA). Fourier‐transform infrared (FT‐IR) spectra were recorded on an IR spectrophotometer (Nicolet iS20, Thermo Fisher Scientific, USA) in the range of 4000–400 cm^−1^. Thermogravimetric analysis was performed on a TGA‐DSC thermal analyzer (STA 449 F5 Jupite, Germany) to obtain TGA and DSC curves. The thermal analysis was conducted in an alumina crucible under argon atmosphere.

### 4.7 Electromagnetic Parameter Measurement

For EMW absorption performance measurements, the samples were uniformly mixed with paraffin wax and pressed into concentric ring‐shaped specimens with an inner diameter of 3.04 mm and an outer diameter of 7.00 mm. The electromagnetic parameters were measured over the frequency range of 2–18 GHz using a vector network analyzer (VNA) based on the coaxial line method. The reflection loss (*R*_L_) values were calculated according to the equations *Z*_in_ = *Z*_0_(*µ*_r_/*ε*_r_)^1/2^tanh[*j*(2*πfd*/*c*)(*µ*_r_ *ε*_r_)^1/2^] and *R*_L_ = 20log_10_ǀ(*Z*_in_−*Z*_0_)/(*Z*_in_+*Z*_0_)ǀ [53, 54], where *Z*_in_ and *Z*_0_ are the input impedance and free‐space impedance, respectively. *c* is the light velocity, *f* is the frequency, and *d* is the absorber thickness.

## Author Contributions

**Zhanming Wu**: methodology, software, investigation, data curation, writing – original draft, writing – review and editing. **Chi Yu**: conceptualization, methodology, software, writing – review and editing. **Nuohua Xie**: project administration, writing – review and editing, methodology. **Xiaojun Zeng**: conceptualization, supervision, funding acquisition, project administration, resources, writing – review and editing, writing – original draft. **Yanfeng Gao**: writing – review and editing, conceptualization, project administration. **Yu‐Nan Tan**: methodology, software, writing – review and editing.

## Conflicts of Interest

The authors declare no conflicts of interest.

## Supporting information




**Supporting File**: advs76556‐sup‐0001‐SuppMat.docx.

## Data Availability

The data that support the findings of this study are available from the corresponding author upon reasonable request.
