# Supplementary material for: Rare‐Earth–Sulfur Surface Modification Enables SiC Ceramics for Low‐Frequency Electromagnetic Wave Absorption in Extreme Environments
Source: Adv Sci (Weinh). 2026 Jul 8:e76556. Online ahead of print. doi: 10.1002/advs.76556 (PMC13344507; doi:10.1002/advs.76556)
Supplement: Supplementary file 1 — Supporting File: advs76556‐sup‐0001‐SuppMat.docx. [file ADVS-9999-e76556-s001.docx]

Supporting Information

**Rare-Earth–Sulfur Surface Modification Enables SiC Ceramics for Low-Frequency Electromagnetic Wave Absorption in Extreme Environments**

Zhanming Wu, Xiaojun Zeng,* Yu-Nan Tan, Chi Yu,* Nuohua Xie, and Yanfeng Gao

In the Debye theory, *ε*″ consists of two parts, as shown in **Equation S1**. The first half is the polarization loss (*ε*_c_*″*), and the second half is conduction loss (*ε*_p_*″*). In the lower frequency range, the EMW absorption materials tend to be dominated by conduction loss, so **Equation S1** can be transformed into **Equation S2**.^[1]^

$\varepsilon^{''}=\frac{\varepsilon_{s}-\varepsilon_{\infty}}{1+\tau^{2}\omega^{2}}\omega\tau+\frac{\sigma}{\omega\varepsilon_{0}}$ (S1)

$\varepsilon^{''}=\frac{\sigma}{2\pi f\varepsilon_{0}}$ (S2)

As can be seen from **Equation S2**, the *ε*″-1/*f* curve can be fitted as a straight line, and the conductivity of the materials in the gigahertz frequency range can be calculated according to the slope of this line. Based on the obtained conductivity, the conduction loss was calculated, while the polarization loss was determined by subtracting the conduction loss from the total *ɛ*ʺ respectively.^[2]^





**Figure S1.** XRD patterns of SiC/RE–S samples.


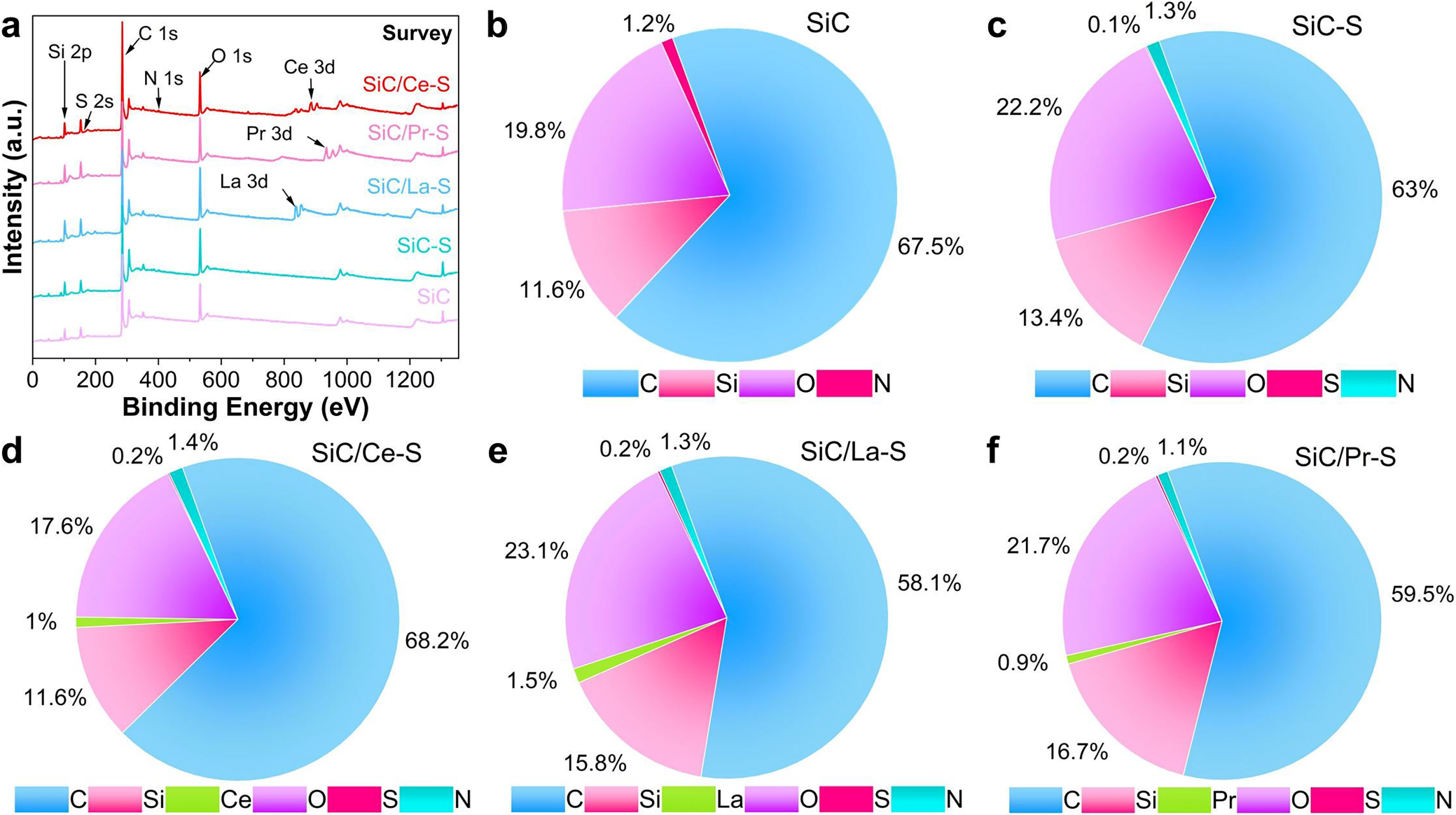


**Figure S2.** (a) XPS survey spectrum and (b-f) elemental content percentages of SiC, SiC–S, and SiC/RE–S.


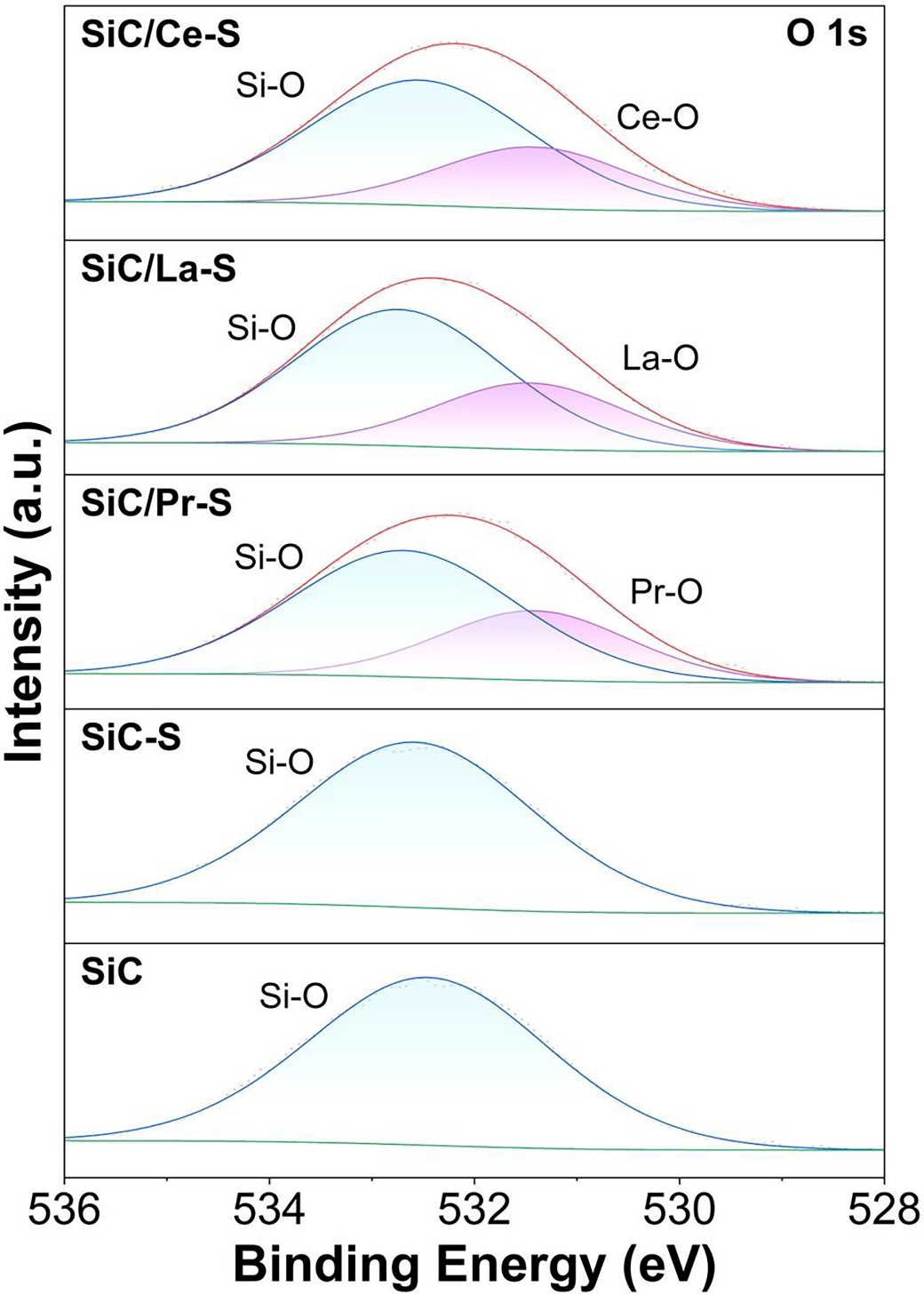


**Figure S3.** High-resolution XPS spectra of O 1s for SiC, SiC–S, SiC/Ce–S, SiC/La–S, and SiC/Pr–S.


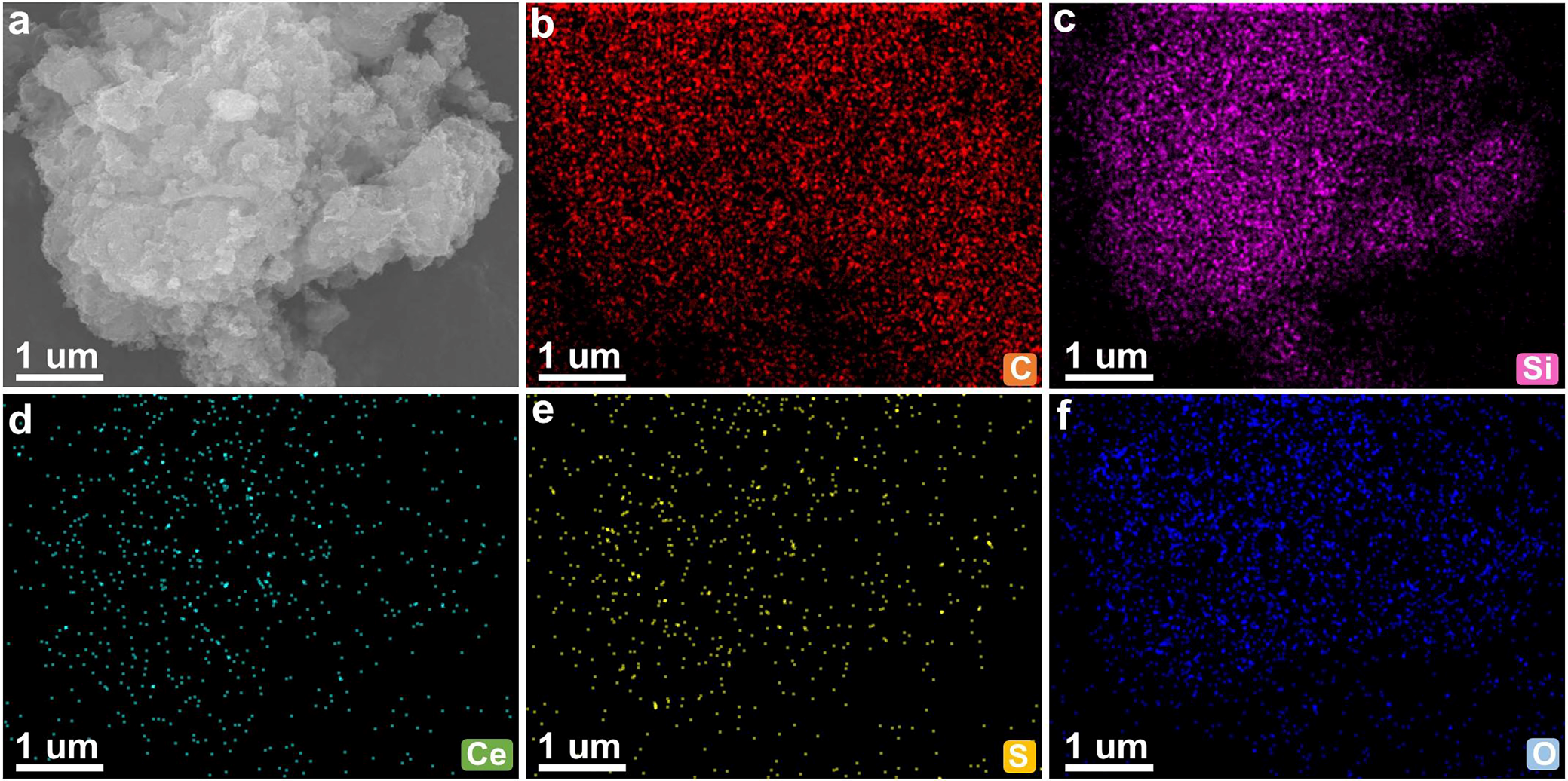


**Figure S4.** Elemental mapping images of SiC/Ce–S.


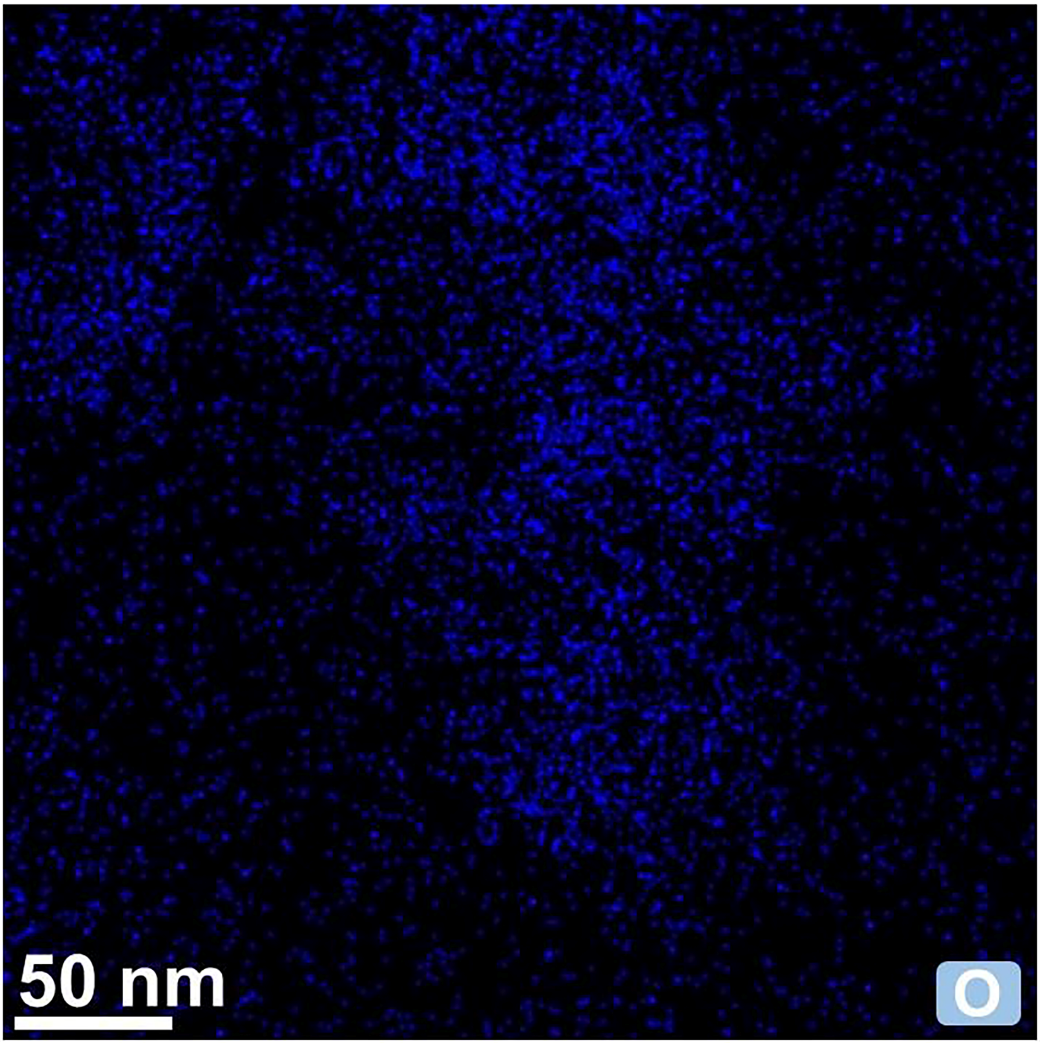


**Figure S5.** Elemental mapping image of O elements for SiC/Ce–S.


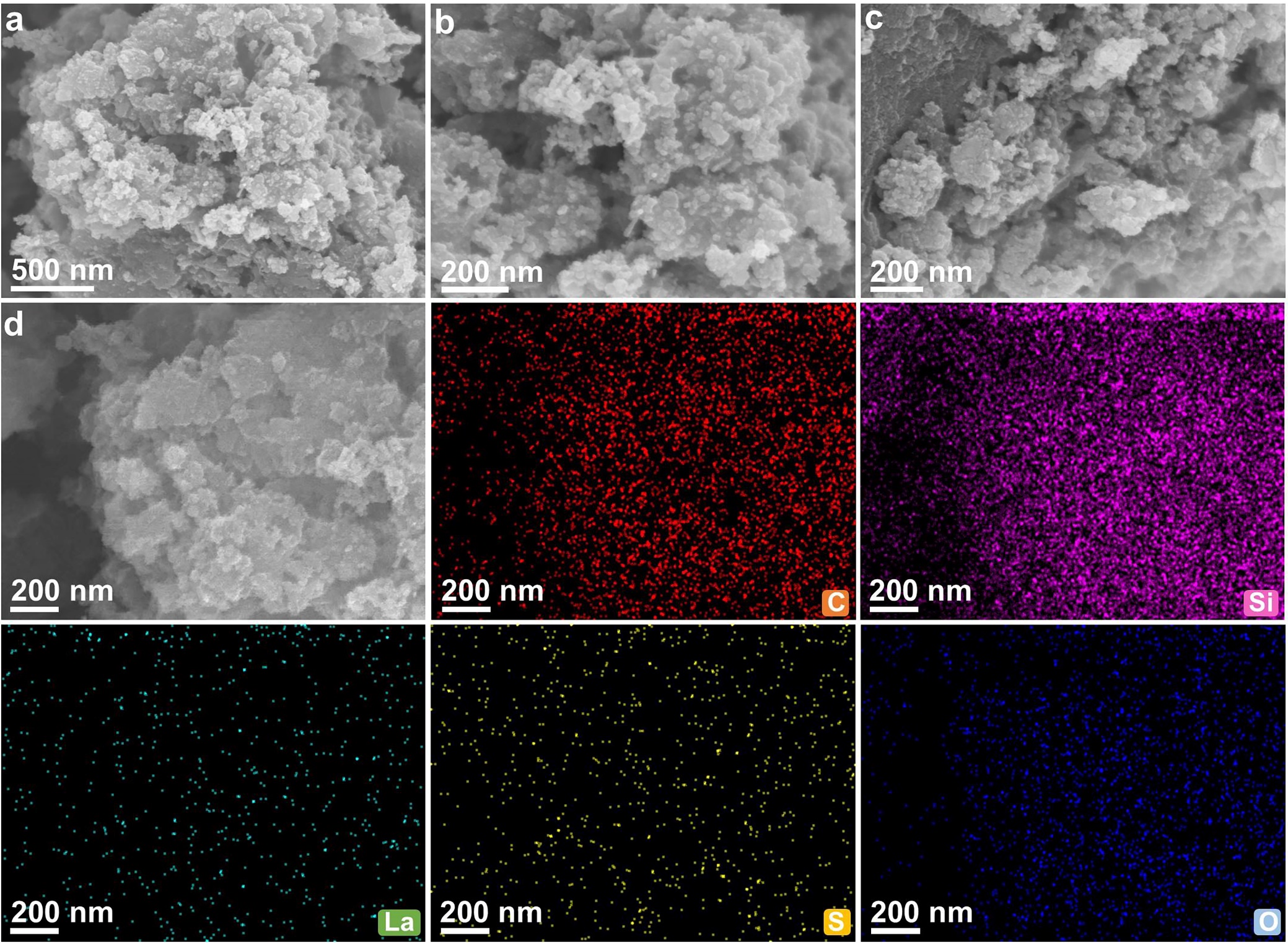


**Figure S6.** (a-c) SEM images and (d) elemental mapping images of SiC/La–S.


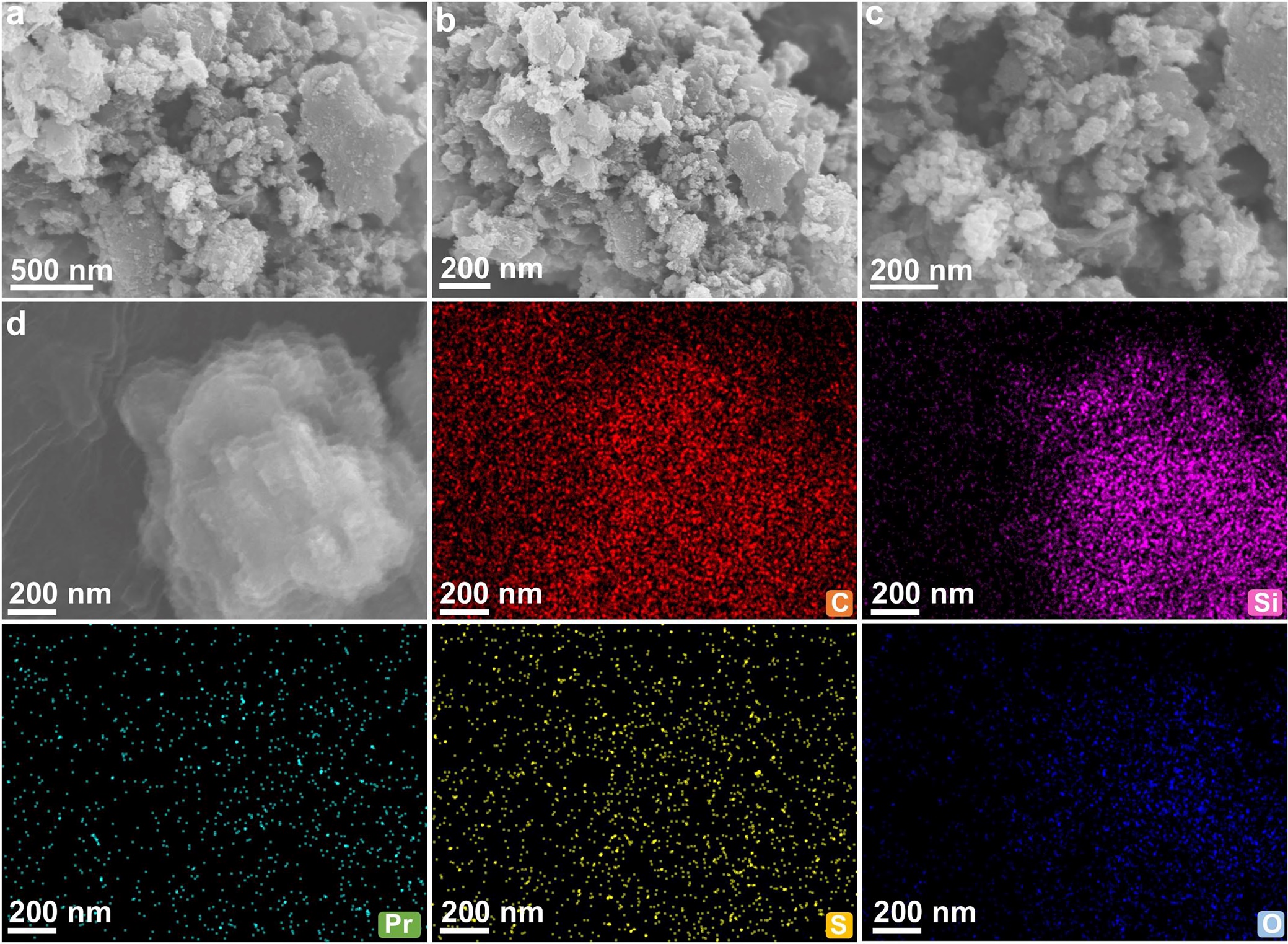


**Figure S7.** (a-c) SEM images and (d) elemental mapping images of SiC/Pr–S.


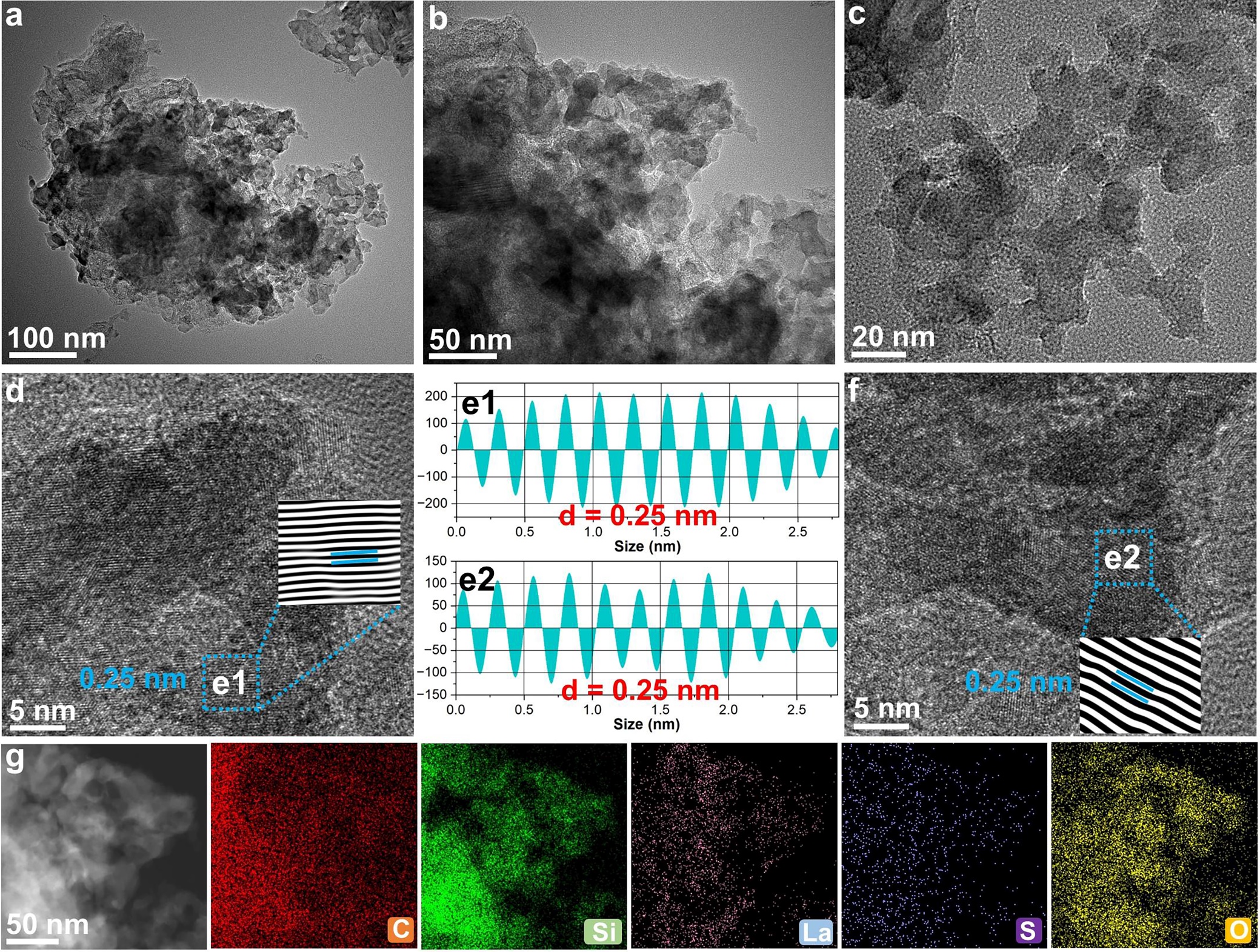


**Figure S8.** (a-d,f) TEM images, (e) lattice spacing measurement, and (g) elemental mapping images of SiC/La–S.


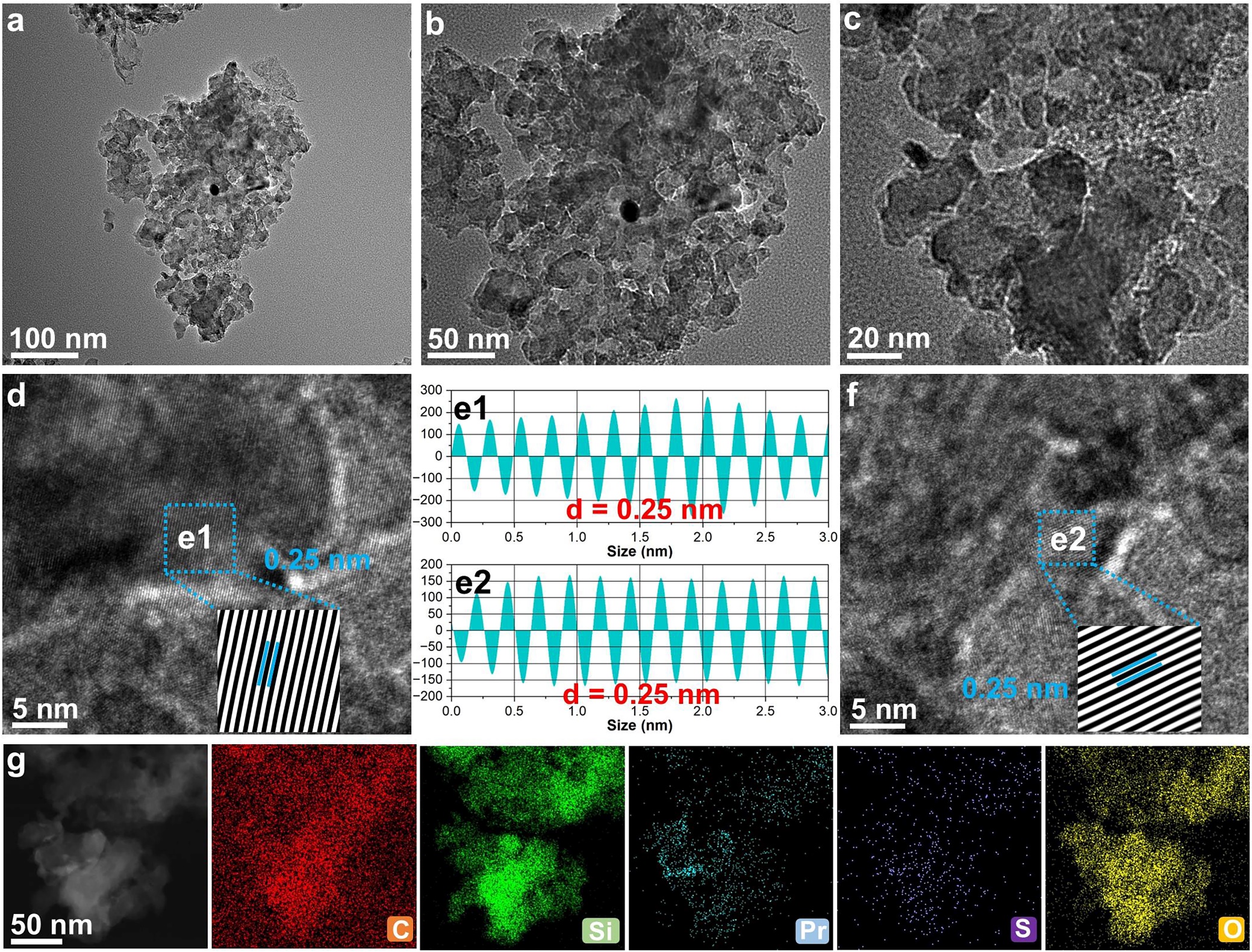


**Figure S9.** (a-d,f) TEM images, (e) lattice spacing measurement, and (g) elemental mapping images of SiC/Pr–S.


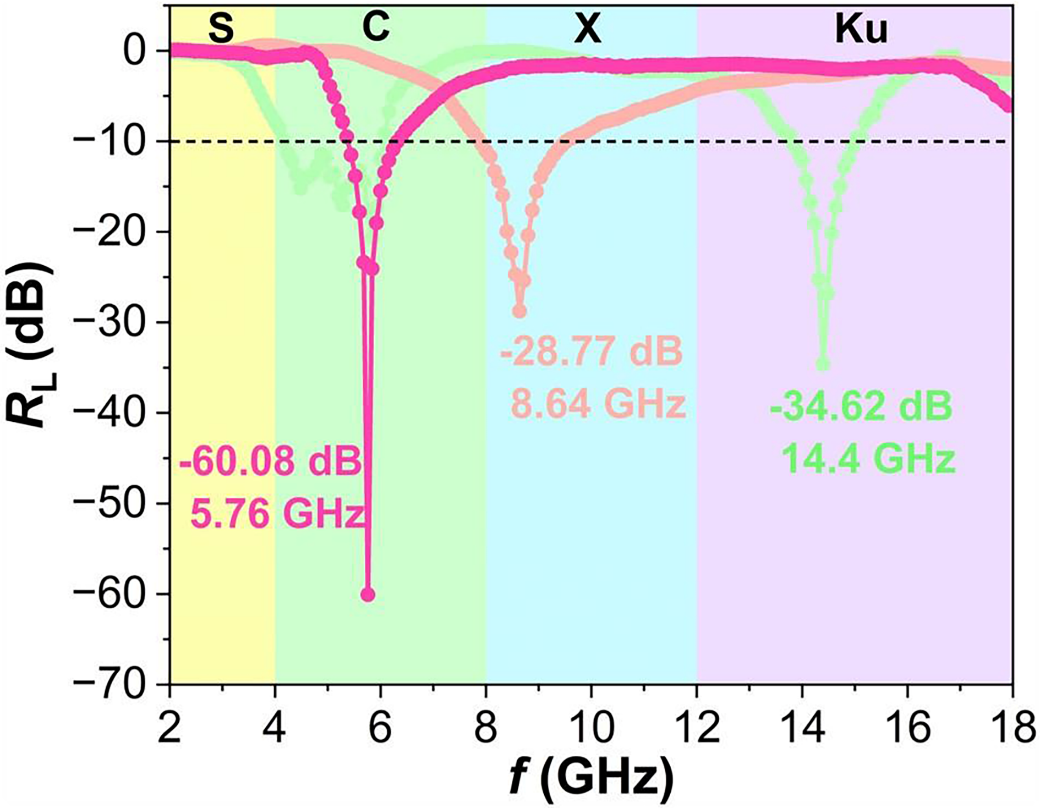


**Figure S10.** 2D *R*_L_ curves of SiC, SiC–S, and SiC/Ce–S.


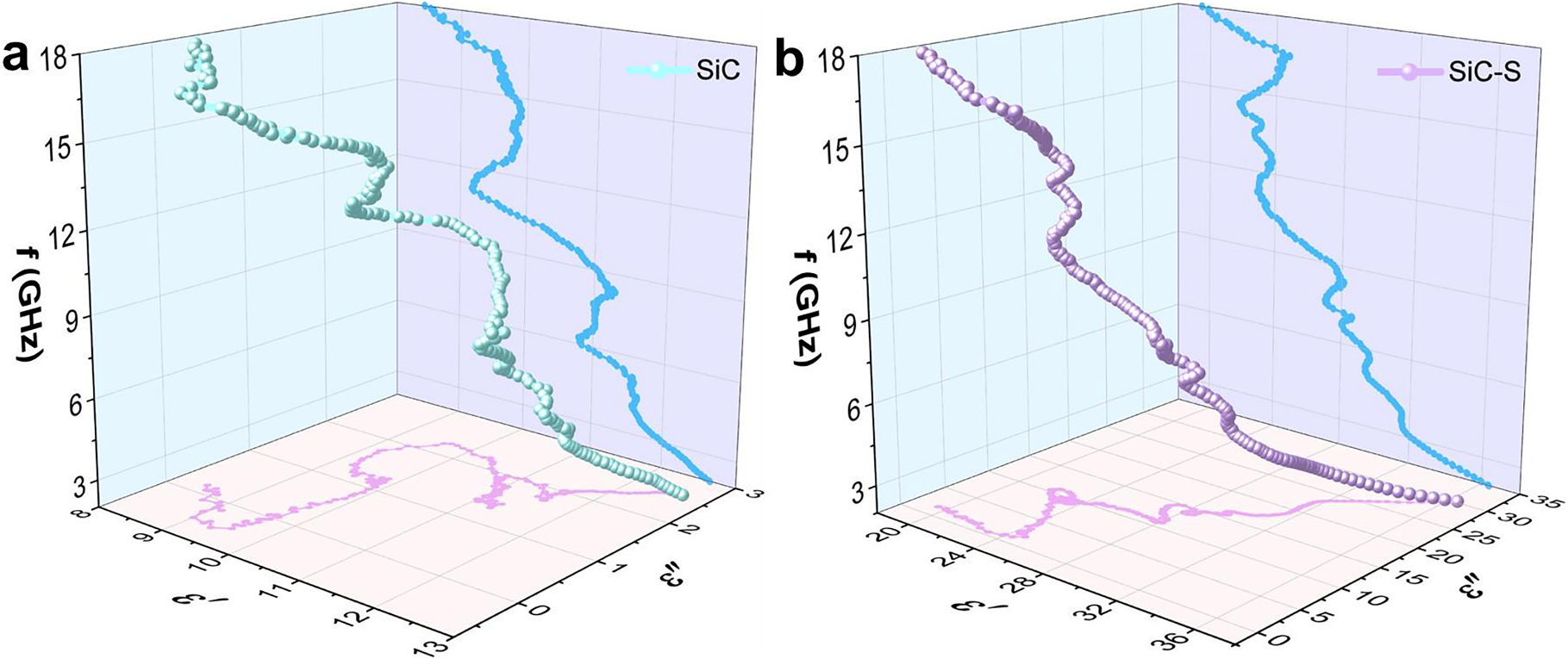


**Figure S11.** Cole‑Cole plots of (a) SiC and (b) SiC–S.


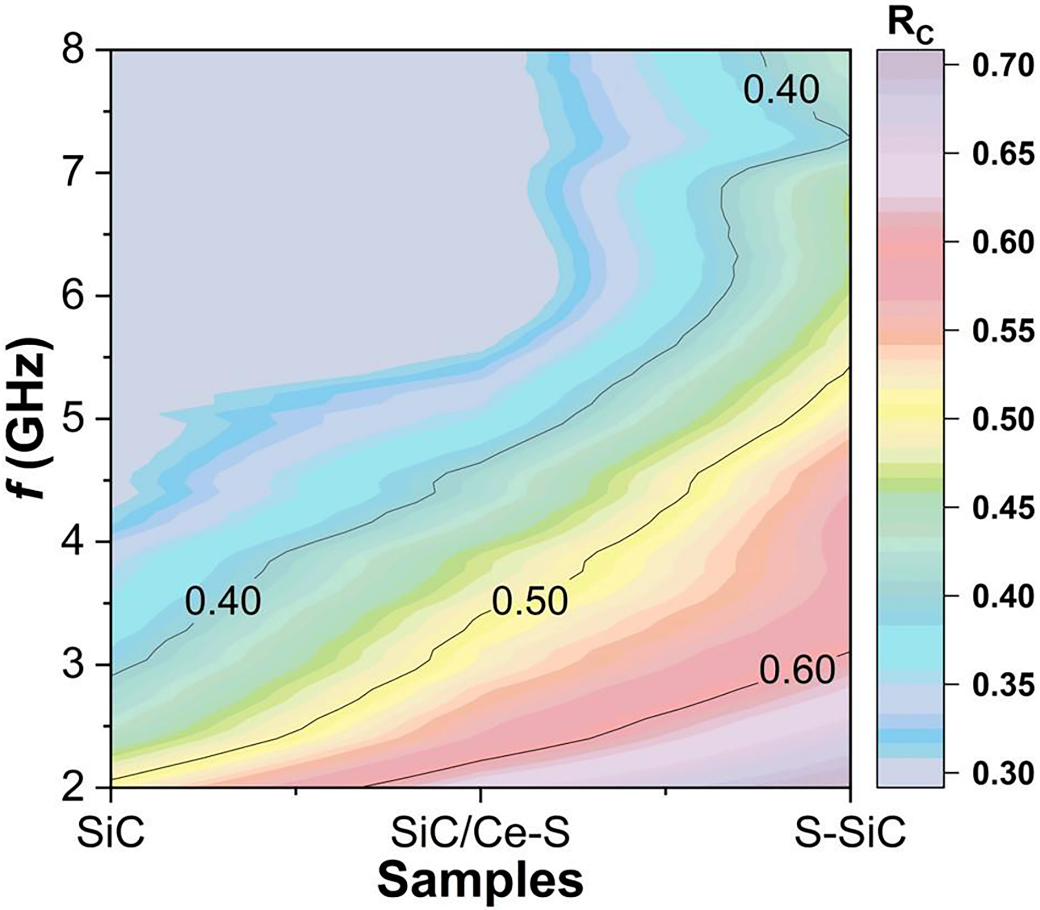


**Figure S12.** Conduction loss proportion of SiC, SiC–S, and SiC/Ce–S.


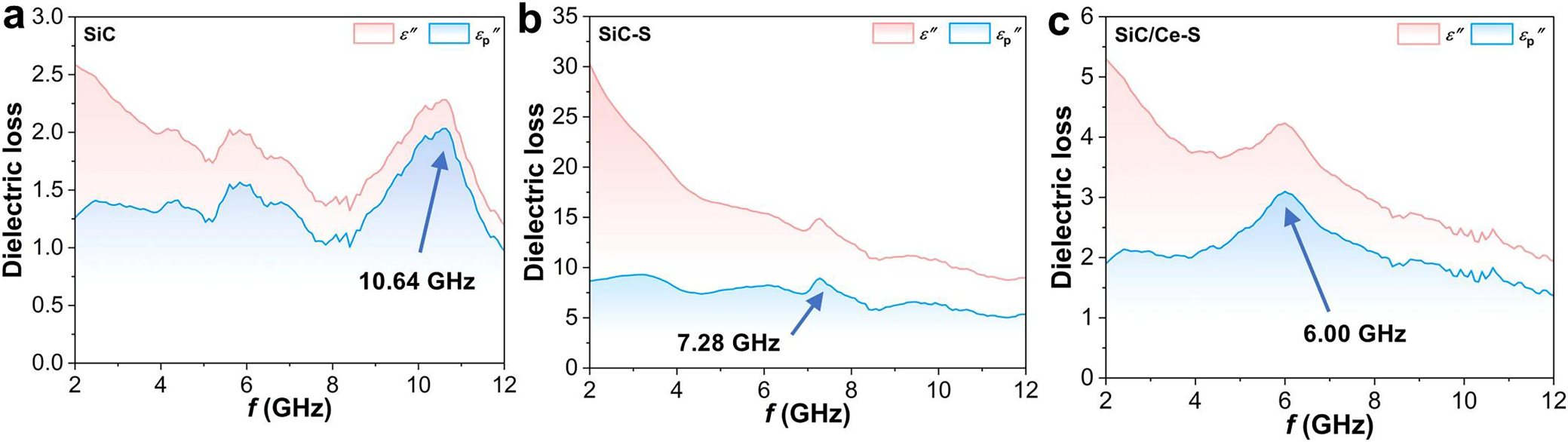


**Figure S13.** Polarization loss and total loss of (a) SiC, (b) SiC–S, and (c) SiC/Ce–S.


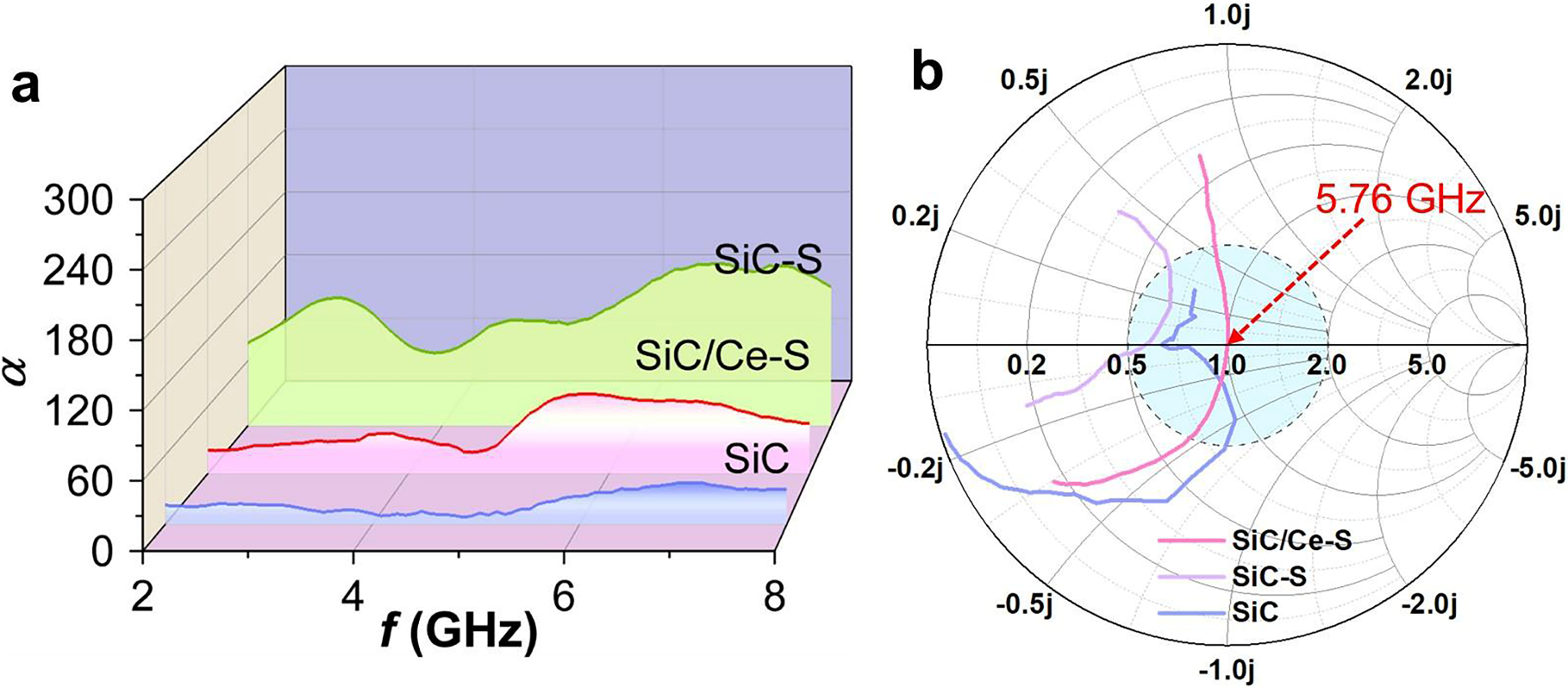


**Figure S14.** (a) *α* and (b) smith chart of SiC, SiC–S, and SiC/Ce–S.


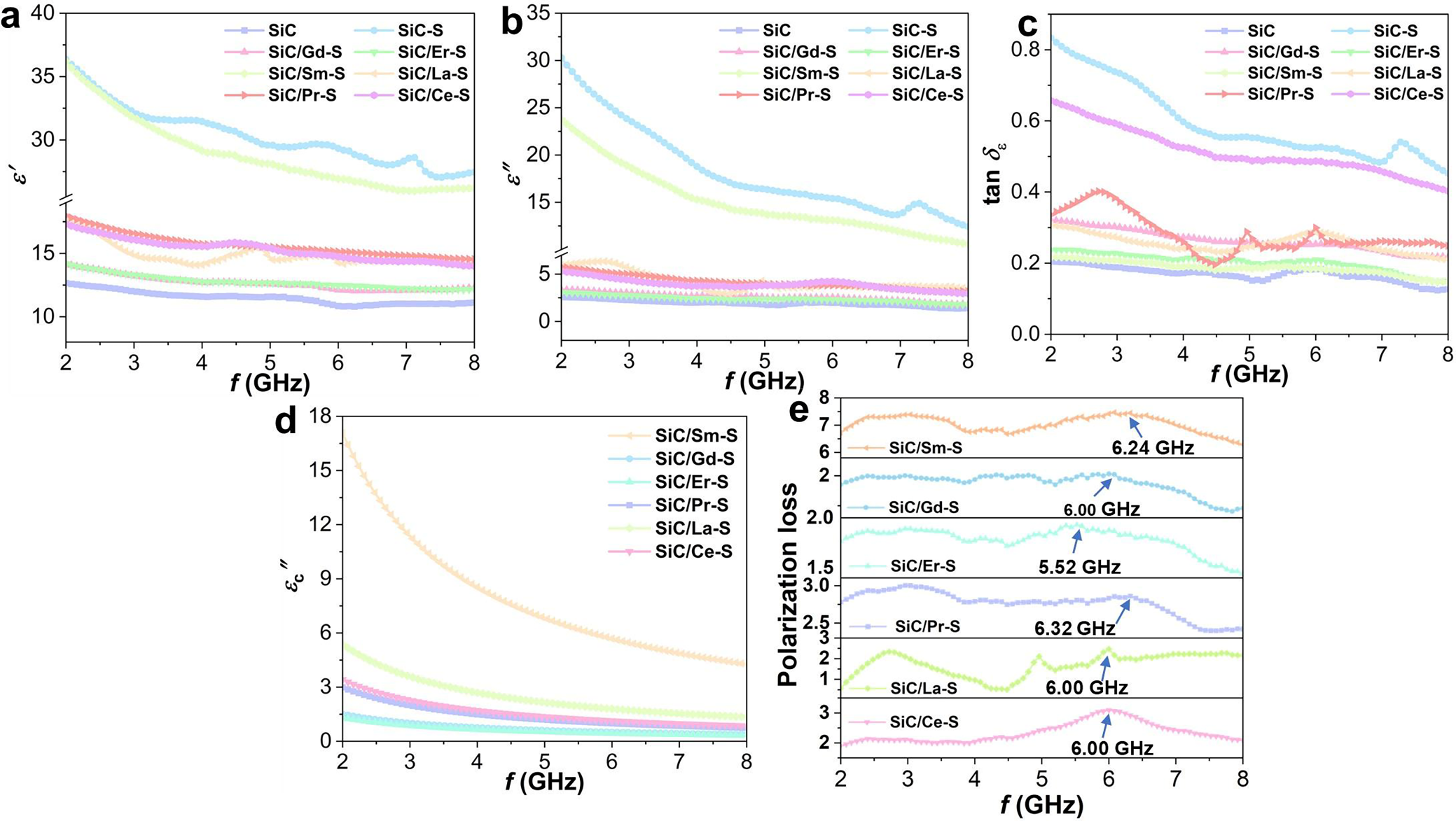


**Figure S15.** (a) *ε′*, (b) *ε″*, (c) tan *δₑ*, (d) *ε_c_″*, and (e) *ε_p_″* values of SiC/RE–S.


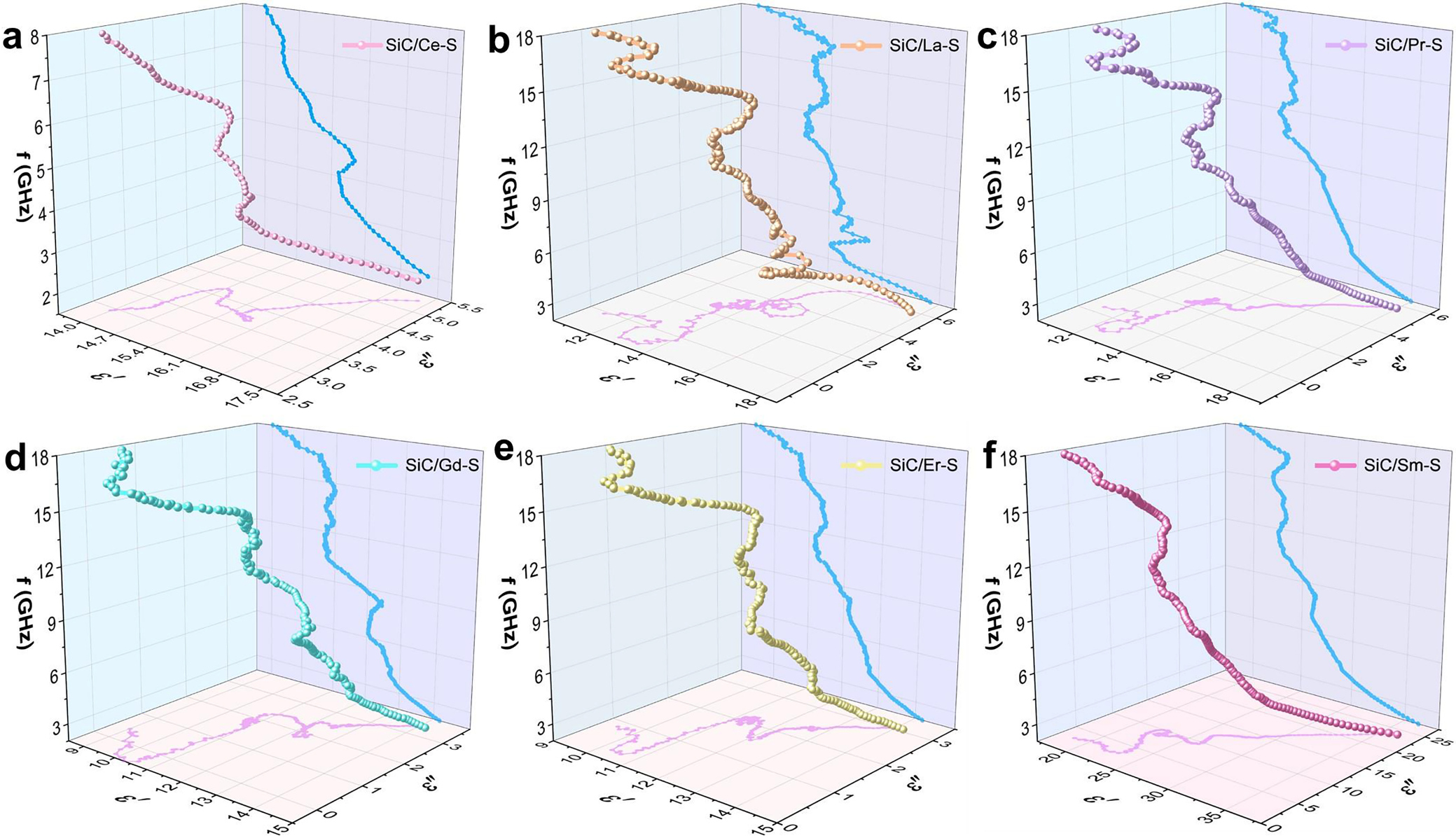


**Figure S16.** Cole‑Cole plots of (a) SiC/Ce–S, (b) SiC/La–S, (c) SiC/Pr–S, (d) SiC/Gd–S, (e) SiC/Er–S, and (f) SiC/Sm–S.


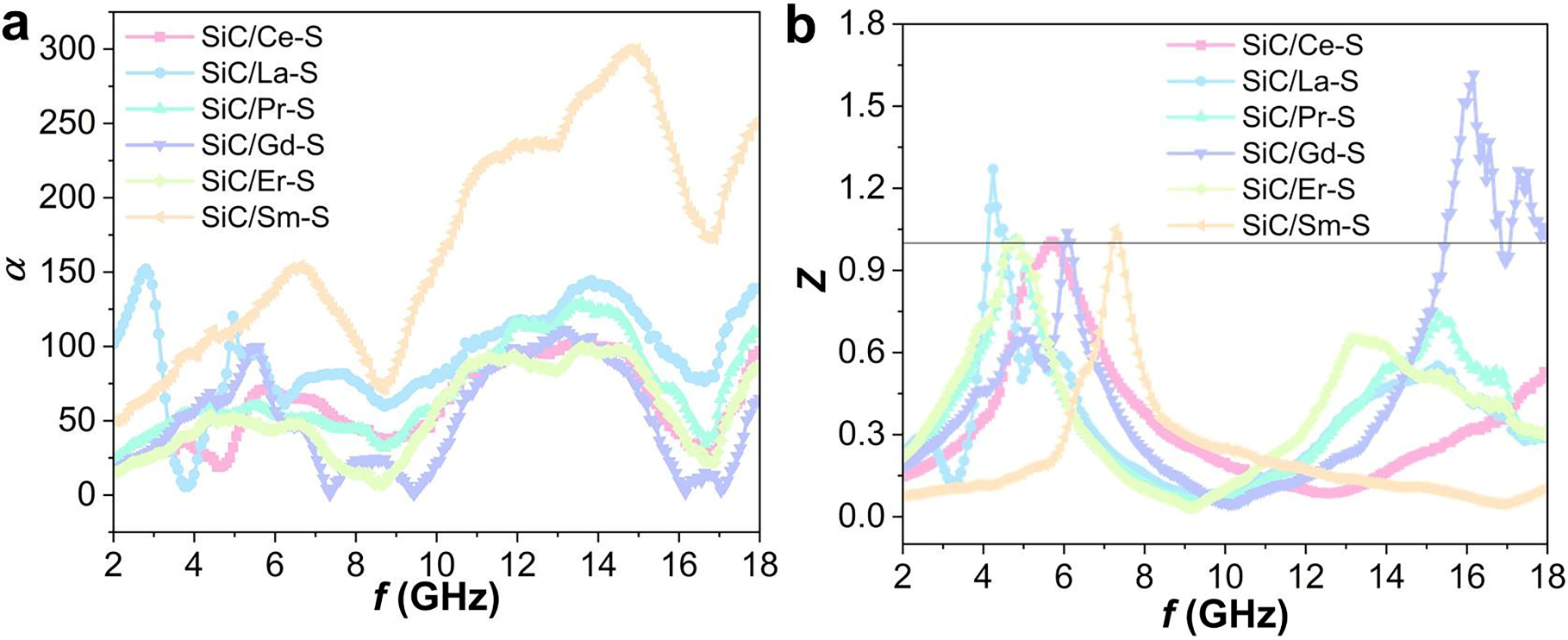


**Figure S17.** (a) *α* and (b) *Z* values of SiC/RE–S.


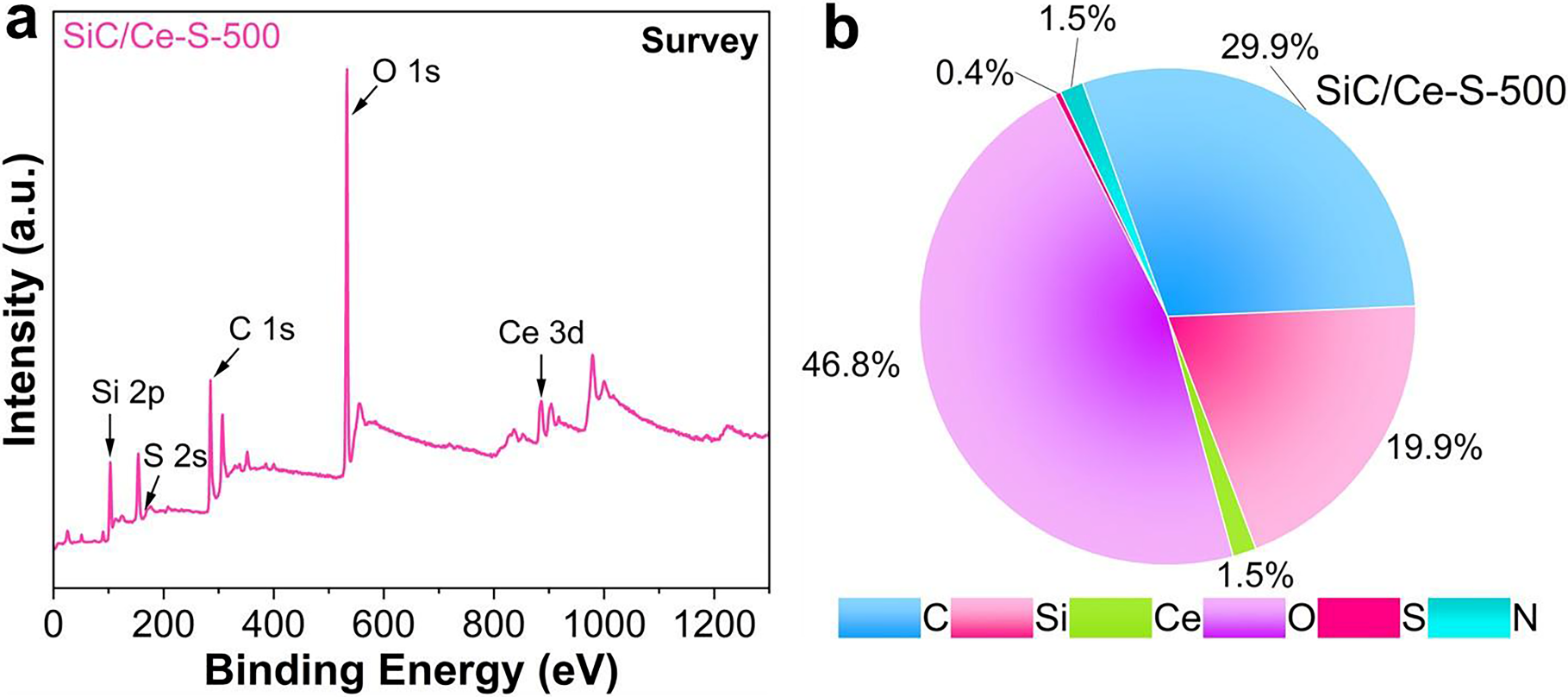


**Figure S18.** (a) XPS survey spectrum and (b) elemental content percentages of SiC/Ce–S‑500.


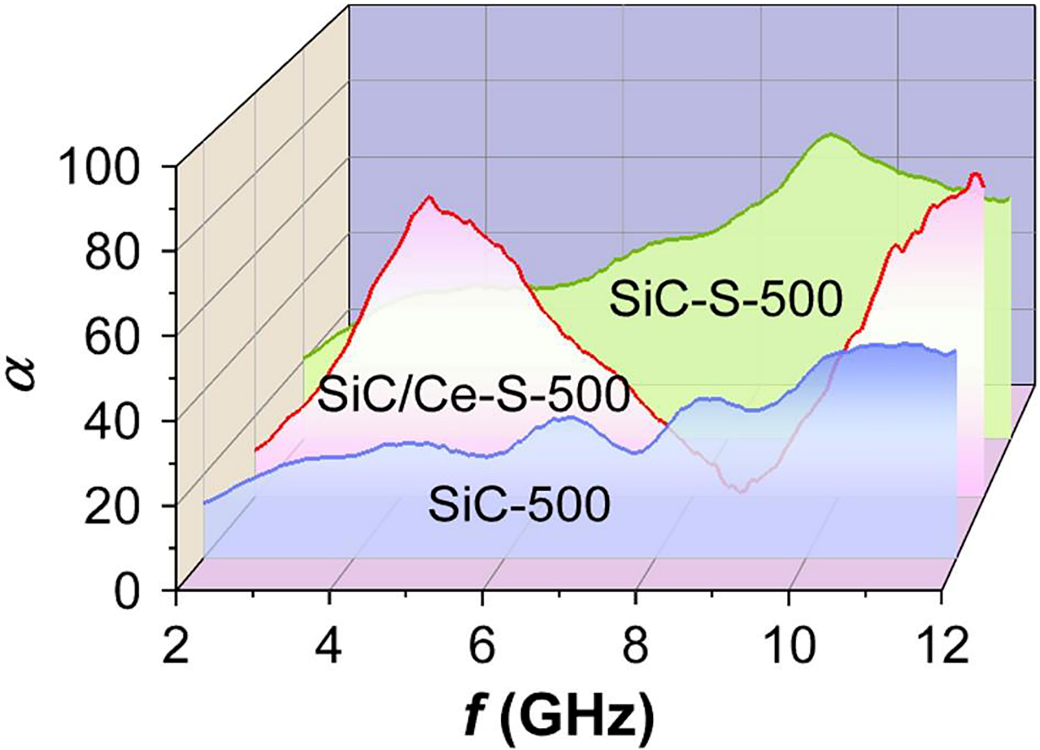


**Figure S19.** *α* values of SiC-500, SiC–S-500, and SiC/Ce–S‑500.


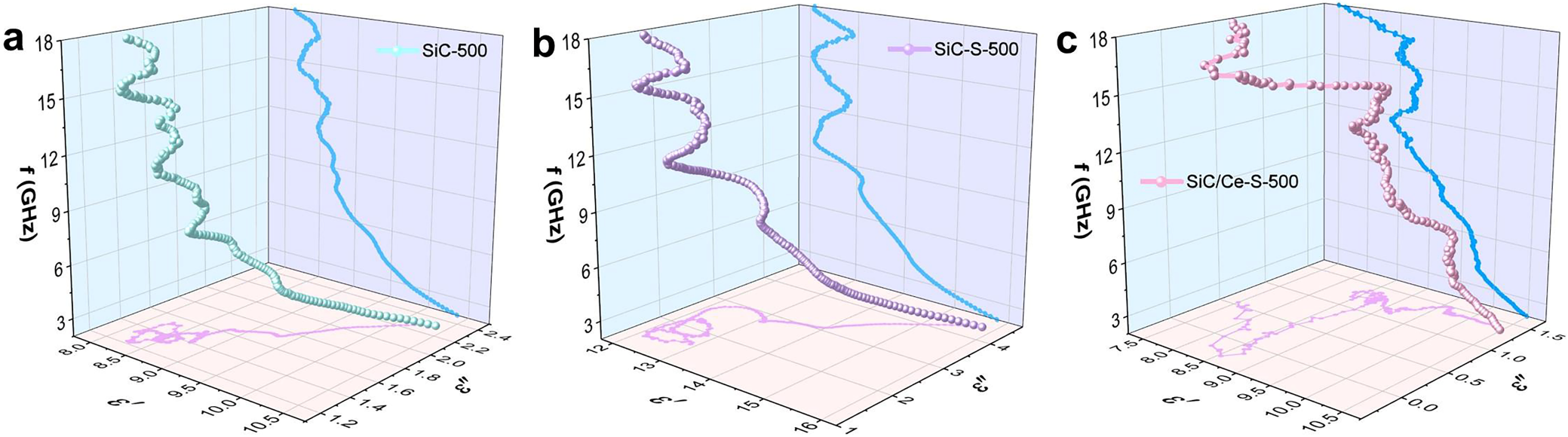


**Figure S20.** Cole‑Cole plots of (a) SiC-500, (b) SiC–S-500, and (c) SiC/Ce–S-500.

**Figure S21.** 3D *R*_L_ plots of SiC/RE–S-800.


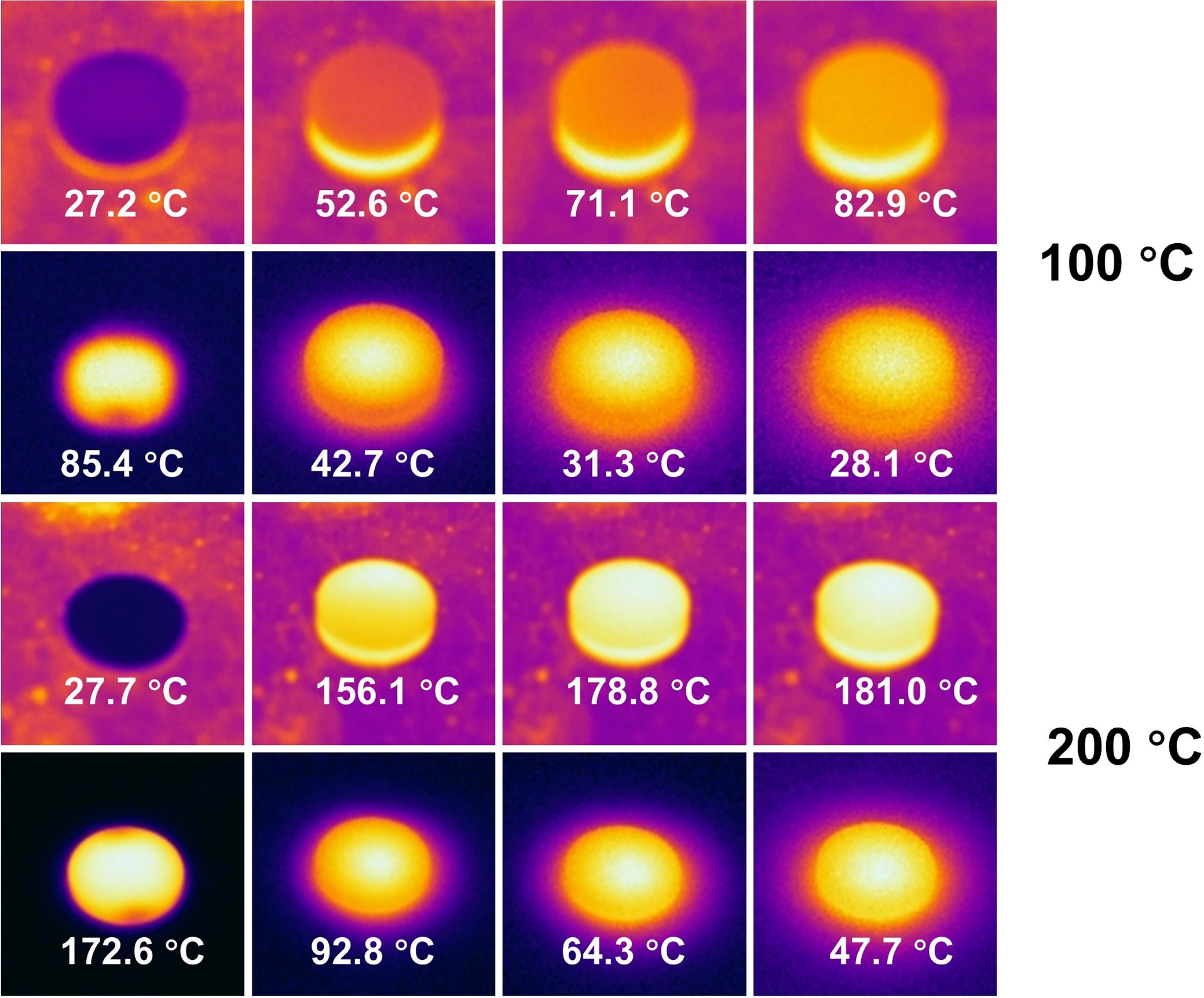


**Figure S22.** Infrared thermal images of the SiC/Ce-S sample during heating and cooling on a 100 °C and 200 °C hot stage.


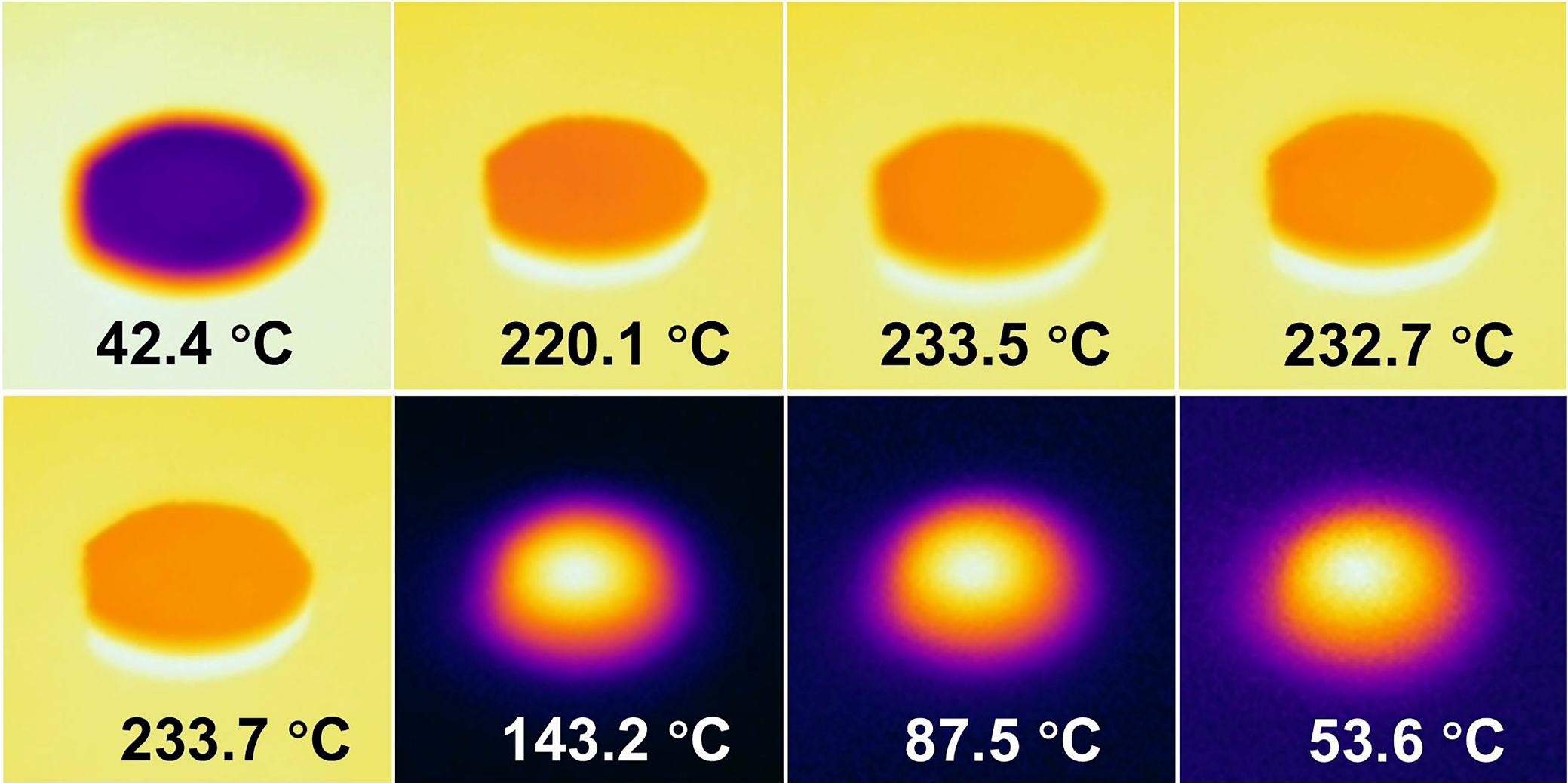


**Figure S23.** Infrared thermal images of the pristine SiC sample during heating and cooling on a 300 °C hot stage.

**Table S1.** EMW absorption performance of SiC/RE–S ceramics and recently advanced SiC-based absorbers.

| Materials | Frequency/GHz | *R*_L_/dB | Ref. |
| --- | --- | --- | --- |
| *α*/*β*-SiC@4MBNS | 13.14 | -52.59 | [3] |
| SiC_f_/ZrC-TiC | 8.96 | -63.50 | [4] |
| SiC@MnO_2_ | 14.50 | -47.96 | [5] |
| SiC_NWs_/RGO | 12.70 | -47.00 | [6] |
| SiC Fibers | 12.85 | -63.34 | [7] |
| Hollow SiC/C | 10.30 | -60.80 | [8] |
| SiC-MTNA | 14.72 | -56.39 | [9] |
| M-TiC/SiC | 9.92 | -67.18 | [10] |
| SiC_NWS_@MnO_2_@PPy | 12.41 | -50.59 | [11] |
| SiC/C-2 | 8.72 | -54.52 | [12] |
| SiC/Ce-S | 5.76 | -60.08 | This work |
| SiC/La-S | 4.56 | -64.05 | This work |

Note: The exact *R*_L_ values and frequency were not presented in some references, thus, those values were dug out according to the *R*_L_-*f* curves.

**References**

[1] B. Ren, Y.J. Jia, L.H. Qi, Q.G. Fu, Y.X. Lin, H. Yu, J.J. Cheng, M.Y. Dai, Q. Song, Y.L. Zhang, X.H. Hou, H.J. Li, Broadband electromagnetic absorption up to 1473 K enabled by dielectric frequency-dispersion engineering in ceramic composites. Nat. Commun. 2026, 17, 318.

[2] K. Zhang, Y. Liu, X. Li, X. Wang, J.X. Liu, X.Y. Liu, All-dielectric ultra-broadband microwave absorbing aerogel with optimized dielectric dispersion via dielectric relaxation time regulation. Adv. Mater. 2025, 37, 2506386.

[3] Y. Xia, M.X. Wu, J.T. Huang, R. Huang, D.H. Ding, J. Yin, C.M. Xu, H.Y. Yang, Q.F. Wan, L.Y. Wang, B.L. Liang, Z.C. Lu, R.Y. Luo, Facilitating structural strengthening and electromagnetic wave absorption functionalization of dual-phase SiC ceramics via MBNS-dominated multiphase reinforcement strategy. J. Adv. Ceram. 2026, 15, 9221234.

[4] J.Q. Liu, C.Y. Li, H.B. Ouyang, T.Z. Shen, S. Zhang, X.N. Wang, X.G. Niu, Multifunctional SiC_f_/ZrC-TiC ceramic foam with outstanding thermal stability: synergistic broadband electromagnetic wave absorption, load-bearing, and thermal insulation. Compos. Part A 2026, 204, 109659.

[5] Y.K. Miao, A.G. Cui, C. Wang, Z.N. Tian, T. Wang, J.Y. Liu, Q.Q. Jia, Z.J. Li, M. Zhang, Regulating oxygen vacancies to enhance dipole and interface polarization for highly efficient electromagnetic wave absorption in SiC@MnO_2_ nanocomposites. Adv. Funct. Mater. 2025, 35, 2503394.

[6] X.T. Li, K. Zhang, J. Wei, Y.B. Zhang, Z. Miao, J.Y. Hou, Y. Yao, Preparation of lightweight layered porous SiC nanowires/RGO composites with excellent electromagnetic wave absorption performance. J. Mater. Chem. C 2025, 13, 7824-7835.

[7] W.F. Kang, Y. Shen, T.Y. Yang, Z.Q. Zhao, Y.Z. Gou, Multifunctional submicron SiC fibers for extreme environments: superior electromagnetic absorption and high-temperature performance. Adv. Funct. Mater. 2025, 35, 2415432.

[8] L.X. Gai, Y.H. Wang, P. Wan, S.P. Yu, Y.Z. Chen, X.J. Han, P. Xu, Y.C. Du, Compositional and hollow engineering of silicon carbide/carbon microspheres as high-performance microwave absorbing materials with good environmental tolerance. Nano-Micro Lett. 2024, 16, 167.

[9] B.J. Zhang, W.X. Zhi, J.S. Duan, L.F. Zhang, F.R. Liang, C. Ma, Mechanically robust SiC aerogel with both electromagnetic absorption and pollutant adsorption via microtube/nanowire structure design. J. Adv. Ceram. 2025, 14, 9221181.

[10] C. Tang, B. Han, L. Dong, M. Liu, J. Ye, X. Yang, J. Meng, W. Wang, Z. Liu, K. Zhao, Y. Tang, In-situ confined transformation of Ti_3_C_2_T_x_ in electrospun SiC fiber matrix for optimized conductive loss and polarization loss. Chem. Eng. J. 2025, 521, 166507.

[11] M. Zhang, L. Zhao, W. Zhao, T. Wang, L. Yuan, Y. Guo, Y. Xie, T. Cheng, A. Meng, Z. Li, Boosted electromagnetic wave absorption performance from synergistic induced polarization of SiCNWs@MnO_2_@PPy heterostructures. Nano Res. 2023, 16, 3558-3569.

[12] B. Xu, Q. He, Y. Wang, X. Yin, Novel accordion-like structure of SiC/C composites for enhanced electromagnetic wave absorption. Carbon 2023, 215, 118470.
